# Improving corrosion depth estimation in buried pipelines using SMOTE-integrated machine and deep learning models

**DOI:** 10.1371/journal.pone.0355187

**Published:** 2026-08-27

**Authors:** Mohamed Khalid AlOmar, Mohammed Majeed Hameed, Osama Khamees Ali, Aleema Ahmad Samir, Tanya Kukreja, Adil Masood, Ali Salem

**Affiliations:** 1 Department of Civil Engineering, Al-Maarif University, Ramadi, Iraq; 2 Upper Euphrates Centre for Sustainable Development Research, University of Anbar, Ramadi, Iraq; 3 Department of Natural and Applied Sciences, TERI School of Advanced Studies, New Delhi, India; 4 Civil Engineering Department, Faculty of Engineering, Minia University, Minya, Egypt; 5 Structural Diagnostics and Analysis Research Group, Faculty of Engineering and Information Technology, University of Pecs, Pécs, Hungary; Hohai University, CHINA

## Abstract

Accurate prediction of corrosion depth in buried pipelines is essential for integrity assessment, reducing inspection and maintenance costs, and managing risk in pipeline transportation systems. Although machine learning (ML) and deep learning (DL) techniques have been widely applied to corrosion prediction, their reliability depends strongly on the quality, representativeness, and balance of the training data. In many previous studies, data imbalance has remained a key limitation, leading to biased models that mainly learn the majority class, underestimate severe corrosion cases, and exhibit reduced overall performance. In this study, a polynomial-based Synthetic Minority Over-sampling Technique (SMOTE) is employed as a data-level solution to alleviate class imbalance and improve model learning. SMOTE is integrated with several predictive models, including a deep learning neural network (DLNN), eXtreme Gradient Boosting (XGBoost), support vector regression (SVR), and multiple linear regression (MLR). The models are developed and evaluated using both the original and the SMOTE-oversampled datasets, and their performance is compared using several statistical metrics. The results show that incorporating SMOTE significantly enhances the predictive capability and robustness of the ML and DL models, attaining a significant reduction of 24.94% in root mean square error (*RMSE*) compared with the corresponding models trained without SMOTE. The DLNN–SMOTE model provides the best performance, achieving a lower *RMSE* (0.624) and a higher correlation coefficient (*R* = 0.940) than other models. These findings demonstrate that SMOTE-enhanced AI models offer an efficient and cost-effective framework for corrosion depth prediction and support more reliable **and sustainable** integrity management of buried pipelines.

## 1. Introduction

Underground pipelines required for transporting oil, gas, and water are critical to the global energy infrastructure, as failures and persistent threats from corrosion pose significant environmental hazards and economic risks [1]. Corrosion is an electrochemical degradation process initiated by compound interactions between the source pipe material and the surrounding soil. Prediction of this primary degradation mechanism is essential for proactive maintenance of the buried systemic pipelines and paramount for risk mitigation [2]. It has been observed that underground pipelines particularly encounter pitting corrosion [3], identified by small yet deep pits, deteriorating the structural integrity of the pipelines, aggravated by environmental factors like soil moisture, chemical soil composition, and temperature fluctuations [4–8]. The complexity and variability of environmental factors in traditional methods of corrosion prediction lead to inefficiencies in resource allocation and safety hazards [2,9].

Improving the reliability of pipeline corrosion prediction can significantly strengthen safety measures, reduce economic losses, and limit environmental harm [10–12]. Industries that handle hazardous materials, especially oil and gas rely heavily on pipeline integrity to protect the environment, making accurate predictive tools essential for early identification of corrosion risks and the prevention of catastrophic failures [13,14]. Artificial intelligence, in this era of big data, has emerged as a transformative technique for modelling complex, multidimensional environmental phenomena, offering superior predictive power compared to traditional methodologies [15,16]. The recent progress of machine learning (ML) and deep learning (DL) has facilitated accurate modeling of complicated interactions between environmental factors, high-dimensional datasets of soil properties, and degradation of materials [17,18]. Furthermore, recent advances in optical sensing technologies have made it possible to detect corrosion depth more accurately without damaging the material [19–21]. These methods provide high-resolution and reliable data that can be effectively used in ML and DL models to improve prediction accuracy. Such approaches can streamline the creation of well-structured and dependable predictive models of sustained corrosion rate prediction, which helps sustain the safety and effectiveness of essential infrastructure [22].

DL has recently emerged as a powerful approach, gaining popularity for its ability to solve complex engineering problems through advanced data-driven techniques [23–25]. These models can effectively extract features from intricate patterns in the data, enhancing the learning process and improving prediction accuracy compared to classical machine learning models. Therefore, these models need to have a well-integrated dataset that accurately represents the target to ensure reliable performance. Deep learning models have successfully captured the complexities of non-linearly correlated datasets [26], addressing intricate engineering challenges and outperforming traditional methods in both accuracy and scalability. However, some DNN models are highly data-intensive and demand substantial high-performance computing resources, escalating costs for training in data-scarce scenarios typical of corrosion datasets [17,27–31]. Contemporary researchers have increasingly utilized ML models like random forest, support vector machines, and gradient boosting against DL architectures such as convolutional neural networks (CNNs) and long short-term memory (LSTM) networks for pipeline corrosion prediction as well as for estimating corrosion rate [18,32,33].

DL-enhanced finite element analysis has been used for stress corrosion cracking prediction [17], outperforming conventional methods in capturing nonlinear interactions [34]. Extreme gradient boosting and dense neural networks have been deployed for corrosion rate estimation in cooling water pipelines, achieving prediction errors [18,35]. Gradient boosting also excelled in predicting pipe failure rates in heating networks by incorporating factors like soil corrosivity and historic incident records [36]. Several other ML models, including Support Vector Machine Regression (SVR), Multivariate Adaptive Regression Splines (MARS), and Extreme Learning Machine (ELM), are adept at managing intricate non-linear datasets prevalent in hydraulic and fluid transport systems [37–39].

Although recent literature explores various AI Models for pipeline corrosion prediction [40–42], some of the existing models suffer from impaired generalization and accuracy in scarce corrosion events due to data imbalance and limited training samples of scarce pitting events [18,36]. The majority of the work implemented for estimating corrosion rates relied on real-world data, entirely neglecting synthetic data generation to augment datasets, leading to underfitting and prediction bias towards more common, non-critical incidents. To enhance the model’s robustness in data-imbalanced, low-training-sample environments, data augmentation and synthetic data generation emerge as vital. However, significant risks and limitations are involved when handling synthetic data; for instance, oversampling may introduce falsified instances in the dataset, misleading the model to learn artificial patterns and degrading natural phenomena, thus increasing the risk of overfitting. The benefits of synthetic data are highly dependent on the generation method, data modality, and mixing ratio with real data. Therefore, concentrating on specific subsets of data particularly critical and extreme values, and systematically increasing their representation in the data used by artificial intelligence models can improve the accuracy of corrosion prediction in buried pipelines. In this case, the reliability assessment plays a crucial role in evaluating predictive models, as it allows their performance to be systematically interpreted. Moreover, it helps determine the optimal amount of synthetic data needed to improve the predictive accuracy of models that typically operate as “black boxes,” offering limited insight into the factors driving corrosion risk.

The lack of experimental samples that accurately represents the target limits the ability of AI models to provide accurate estimates due to imbalances in the data samples. Consequently, researchers have explored various approaches to compensate for these shortcomings, aiming to enhance data quality and improve the prediction learning process. Synthetic augmentation using Conditional Generative Adversarial Networks (CTGAN), and Generative Adversarial Networks (GANs) generate realistic corrosion growth data [43,44], enhancing model performance compared to training solely on real data. However, these approaches are complex and may carry the risk of overfitting due to the introduction of artificial patterns, particularly when careful attention is not given to mixing ratios. Moreover, there is often only a limited improvement in accuracy with this method, as these sample-generating approaches require careful adjustments due to their complexity. While prior studies have effectively used synthetic data generation to augment models for corrosion prediction, applications of targeted augmentation techniques like SMOTE remain limited, particularly for pitting corrosion in pipelines [31,45,46]. Also, because SMOTE is less complex than previous methods such as GANs, and CTGAN, its application in the field of corrosion, particularly when integrated prediction models to enhance performance, has been notably limited in the existing literature [18,47]. Thus, the current research aims to pioneer the integration of SMOTE with ML and DL models to enhance accuracy and generalization while mitigating the impact of low sample regimes, which can create imbalances in the training dataset [28,48]. This research also conducts a comprehensive analysis to prioritize the input environmental parameters (e.g., soil moisture, potential, bicarbonate) that affect pitting depth, providing valuable insights that are often lacking in black-box synthetic methodologies [31,49]. The outlined objectives of the study are:

1. To apply SMOTE for synthetic minority oversampling in imbalanced corrosion datasets, train both ML and DL models
2. To evaluate the performance improvements achieved by SMOTE by comparing models trained with and without SMOTE using multiple statistical metrics.
3. To conduct a sensitivity analysis for identifying preeminent factors on depth corrosion, optimising feature selection for model robustness.

## 2. Data collection

This study employed a case study methodology, utilizing a well-compiled dataset sourced from openly available literature on pitting corrosion in buried pipelines. The dataset was thoroughly detailed in a technical note by previous research [50]. The data collected in this study are provided in the Appendix of the Supplementary Information. Notably, the collected samples used in this work were only compiled from field measurements collected at multiple pipelines dig sites over a three-year period. The core outcome to examine is the maximum pit depth (*d*_*max*_), the largest observed corrosion pit along the surface of a pipeline. Deeper pits are more critical than any other source of buried pipe failure, as they reflect the most destructive localized loss of metals and frequently determine the overall structural health and life in buried pipelines [51]. The data set consists of 259 field measurements, which encompass both pipeline properties and the surrounding soil conditions. These variables encompass a great deal of factors that are known to affect corrosion behavior:

- Redox potential (rp): A metric that reflects whether a soil environment will support oxidation or reduction and consequently affect corrosion responses.
- Soil pH: reflects how acidic or alkaline the soil is; the lower the pH, the faster the corrosion.
- Pipe-to-soil potential (pp): An important factor for assessing the effectiveness of cathodic protection systems because it compares the voltage between the pipeline and soil.
- Soil resistivity (re): A key indicator of how naturally electrical current flows through the soil, and low resistivity is a known indication of increased corrosion risk.
- Water content (wc): Indicates soil moisture, which supports ionic movement in soil and accelerates the electrochemical activity.
- Bulk density (bd): It impacts the porous nature of your soil and its permeability, which can influence the velocity at which corrosive agents travel up to the pipeline surface.
- Chloride (cc), bicarbonate (bc), and sulfate (sc) ions: Main forms of active dissolved ion, which affect corrosion processes. Chloride in particular is notorious for causing corrosion pitting.
- Pipeline age (t): Is expressed in years which show how much time the pipeline has been exposed to underground conditions.
- Type of coating (ct): A categorical variable reflecting the protective coating put on the pipe and the performance of the coating.

All together, these variables provide a detailed account of environmental and operational factors that influence underground corrosion. For model modeling, the complete dataset was randomly split out, and 75 percent of the data was used for model training and 25 percent for testing. This division of data gives an indication that the models have been tested on genuinely unseen data, which ensures a fair estimation of their predictive abilities.

**Fig 1:**
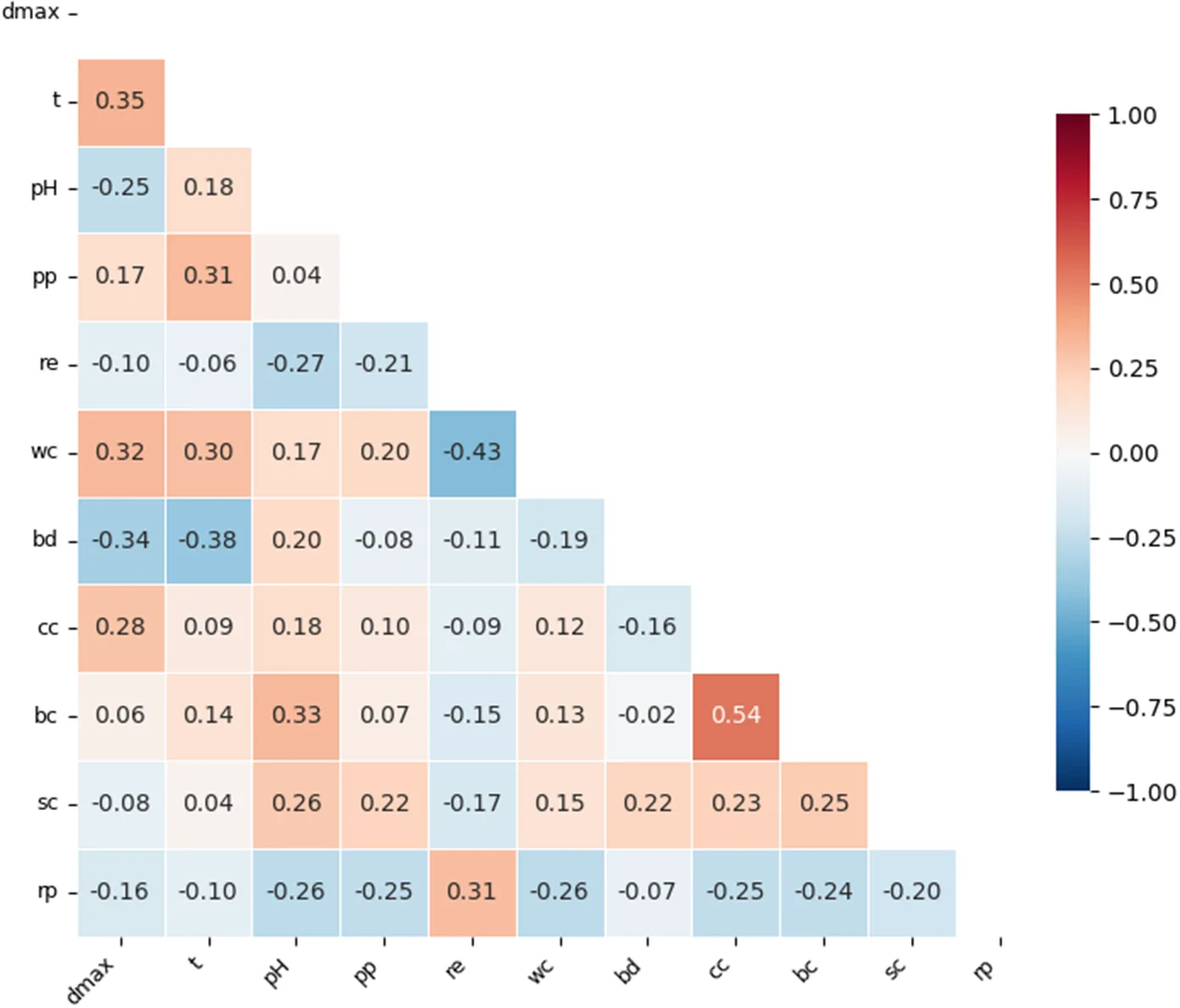
Correlation matrix of the selected corrosion parameters.

In this work, the dataset did not contain any missing value; therefore, all available experimental samples were retained for the analysis. Prior to applying ML-based models, the input variables were scaled to a range between 0 and 1 using a standard min–max normalization approach based on the training data only. No outliers were removed, as the dataset was intentionally used in its original form to evaluate model performance under realistic conditions. It is worth noting that these extreme values reflect cases of severe corrosion and are essential for evaluating how well the models can capture such critical behavior and ensure reliable predictions. The training data as reported in Table 1 were sufficient to cover all the significant natural variations that are seen in a few soil parameters, including soil resistivity (re), chloride (cc), sulfate (sc), and redox potential (rp), all of which can vary in natural subsurface conditions. Mean maximum pit depth (d_max_), the target variable, varied widely in both subsets. Mean d_max_ values were varying from 1.635 to 1.840 mm, while the skewness of 2.455 to 2.705. The strong positive skew in both training and testing samples (high positive skew in both sets vs. 0) suggests that occasionally very deep pits exist, as they are essential indications of severe corrosion and critical in learning from the model. According to Fig 1, d_max_ mainly depends on several independent variables, particularly t, bd, w𝑐, c𝑐, and pH. These variables exhibit strong linear correlations with d_max_, indicating their significant influence in a linear system. In contrast, the remaining variables show lower correlation coefficients (*R*), suggesting a weaker linear effect on d_max_.

**Table 1:** The statistical description of the data used.

Data set	Parameters	Minimum	maximum	mean	std	skewness
Training	t	5.000	42.000	21.687	8.161	0.911
	pH	4.160	7.620	5.974	0.809	−0.017
	PP	−1.950	−0.470	−0.865	0.227	−0.889
	re	3.800	243.600	52.958	49.322	1.758
	wc	11.400	37.200	22.676	5.912	0.221
	bd	1.120	1.480	1.299	0.075	0.225
	cc	3.520	128.500	29.072	28.814	1.850
	bc	5.100	59.350	13.451	8.522	2.950
	sc	11.300	958.900	152.268	143.311	3.175
	rp	11.000	348.000	182.583	87.971	0.036
	*d*_*max*_	0.410	8.560	1.840	1.662	2.455
Testing	t	6.000	42.000	20.795	8.492	0.726
	pH	4.750	9.360	6.277	0.995	0.784
	PP	−1.440	−0.490	−0.884	0.222	−0.206
	re	1.900	161.200	40.892	43.365	1.555
	wc	15.200	38.900	24.377	5.875	0.256
	bd	1.110	1.490	1.309	0.085	−0.149
	cc	4.960	289.500	33.805	52.731	3.456
	bc	5.550	46.220	14.989	10.846	1.698
	sc	14.900	1370.200	171.965	238.580	3.724
	rp	19.000	309.000	162.726	78.495	0.049
	*d*_*max*_	0.410	8.260	1.635	1.784	2.705

## 3. Methods

Corrosion‍‍‍ of underground pipelines results from soil chemistry, moisture, coating degradation, microbial activity, and subsurface heterogeneity interacting with each other. Based on the findings of research, corrosion behaves randomly and is very difficult to model through traditional linear methods because of significant soil variability [52,53]. Machine learning methods are considered as the most promising alternatives that can provide better performance by capturing the nonlinear relationships between the parameters that cause corrosion [54,55]. However, the biggest problem is that field datasets are extremely unbalanced whereby situations of high corrosion are fewer as compared to those of mild or moderate corrosion, thus lowering model sensitivity and increasing predictive uncertainty [56,57]. In order to counter that, this research uses synthetic oversampling (SMOTER) to enhance the machine learning and deep learning models’ capacity to learn which is a method that has never been used before in the prediction of underground corrosion. The performances of the four predictive models- Deep Learning Neural Network (DLNN), Support Vector Regression (SVR), Extreme Gradient Boosting (XGBoost), and Multiple Linear Regression (MLR) are compared over the original and synthetically balanced ‍‍‍datasets.

### 3.1. Deep Learning Neural Network (DLNN)

Deep learning models have demonstrated strong capability in capturing complex and nonlinear relationships, particularly in corrosion prediction problems where multiple soil and environmental parameters interact dynamically. In this study, a Deep Learning Neural Network (DLNN) is employed to model such nonlinear patterns and improve prediction accuracy. The DLNN employed in this study is made up of dense layers with ReLU activation, which enable deeper feature extraction and better pattern recognition in corrosion data. The latest research shows that deep neural networks exceed the performance of classical models in the case of corrosion caused by changing underground conditions and coating degradation [55]. As a result, DLNN is the first primary deep learning model included to investigate whether its capacity for detecting severe condensation zones is enhanced ‍‍‍by synthetic oversampling.

**Fig 2:**
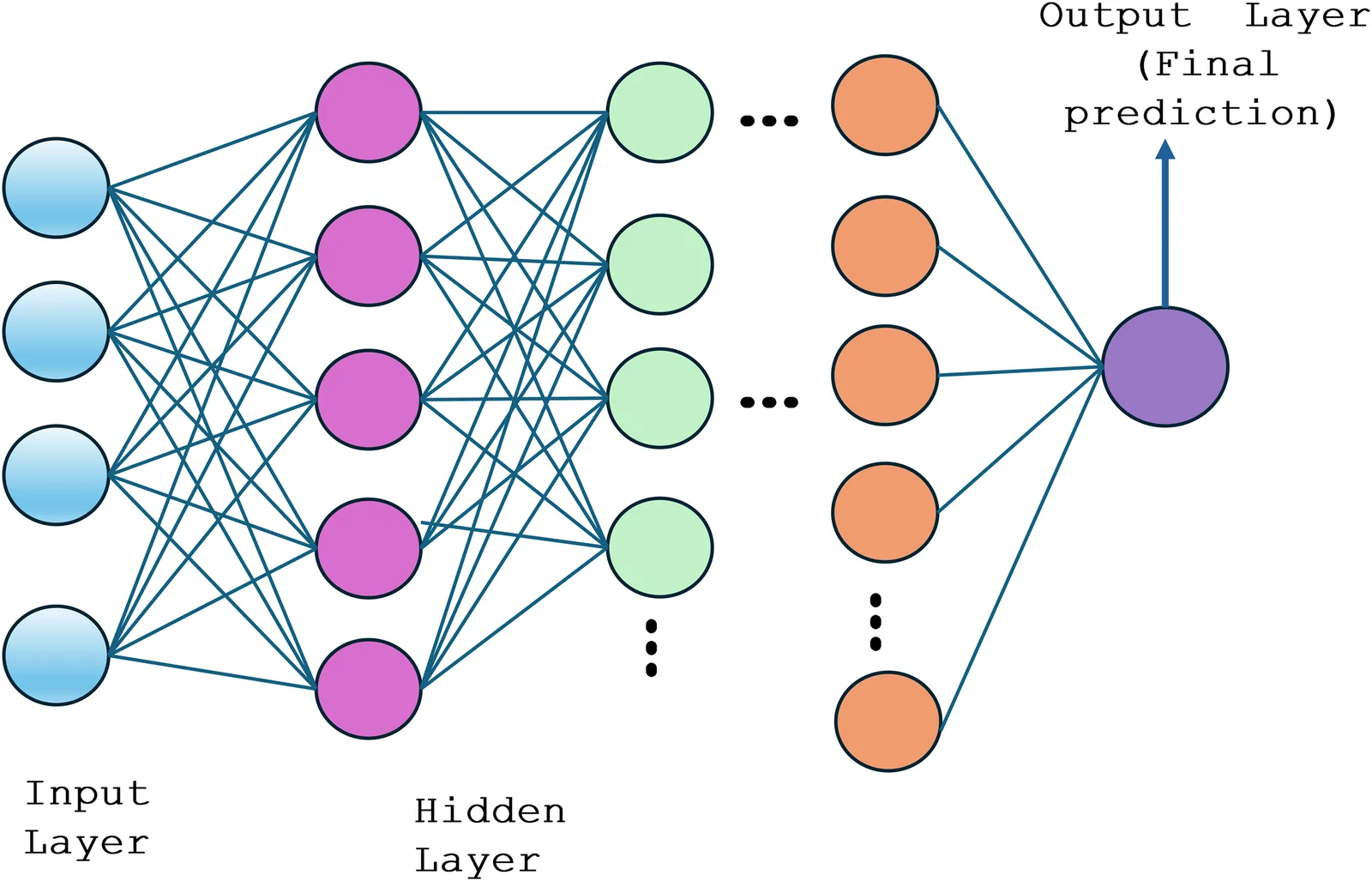
Deep learning structure.

The proposed DLNN adopts a fully connected feedforward architecture consisting of an input layer, six hidden layers, and a single-neuron output layer, as illustrated in Fig 2. The input layer comprises *n* neurons corresponding to the number of selected soil and environmental features, while the output layer produces a single continuous value representing the predicted corrosion depth. The network architecture includes six dense hidden layers with a progressively decreasing number of neurons to facilitate hierarchical feature extraction. Specifically, the number of neurons in each hidden layer is 100–150, 50–100, 30–70, 20–30, 15–25, and 8–15, respectively. The output layer is configured with a linear activation function. This tapered structure enables the model to learn high-dimensional representations in the early layers and gradually transform them into more compact and task-specific features in deeper layers.

Each hidden layer makes use of the Rectified Linear Unit (ReLU) activation function that helps in adding nonlinearity and boosting the model’s capability of recognizing complex patterns. Mathematically the forward propagation through the network is written as:


$$\begin{array}{c} {𝐡}^{\left(l\right)}=\text{ReLU}\left({𝐖}^{\left(l\right)}{𝐡}^{\left(l-\text{1}\right)}+{𝐛}^{\left(l\right)}\right)\; \end{array}$$ (1)

where $h^{\left(l\right)}$ is the output of the hidden layer, $W^{\left(l\right)}\;and\;b^{\left(l\right)}\;$ denote the weight matrix and bias vector, respectively, ϕ(·) is the ReLU activation function. The input vector ${𝐡}^{\left(\text{0}\right)}$ consists of the selected soil and environmental parameters. Model parameters are optimized by using backpropagation to minimize prediction errors. The use of synthetically balanced datasets enhances the network’s capability to learn rare high-corrosion patterns and improves predictive reliability.

Model parameters are optimized using backpropagation to minimize prediction error. The network is trained for 300−1000 epochs with a batch size of 25−25, and a validation split of 0.1–0.25 is employed to monitor generalization performance during training. Notably, the model in this work was trained using the Adam optimizer with mean squared error (MSE) as the loss function. To help reduce overfitting, an early stopping approach was used, with the patience value tuned in the range of 10–30 epochs. Also, the learning procedure was stopped once the validation loss did not improve for the selected number of consecutive epochs, and the model weights with the best validation performance were restored. In this work, a validation split of 20% of the training data was used during training. Overall, the proposed DLNN architecture provides an effective balance between model complexity and predictive performance, making it a reliable framework for estimating corrosion depth in buried pipelines.

To address data imbalance and improve the model’s ability to learn rare high-corrosion cases, the Synthetic Minority Over-sampling Technique (SMOTE) is applied. The DLNN-SMOTE variant uses a sampling ratio in a range of 5–50%, enhancing the representation of under-sampled corrosion scenarios. Furthermore, in this work No explicit regularization techniques is employed such as Dropout or Batch Normalization are incorporated in the model. Instead, overfitting is mitigated through careful architectural design, dataset balancing, and validation monitoring through both testing phases and early stopping.

### 3.2. SVR

The Support Vector Regression (SVR) technique is a great fit for solving environmental and engineering prediction problems. This is especially the case when you have limited, noisy data, with complicated nonlinear relationships. SVR has a solid theoretical basis and lets you regulate the complexity of the model, so it is a very good and reliable method for corrosion prediction tasks [58,59]. In the case of buried pipeline corrosion where there can be noise in measurement data and sudden changes in soil parameters, the argument for SVR is even stronger.

**Fig 3:**
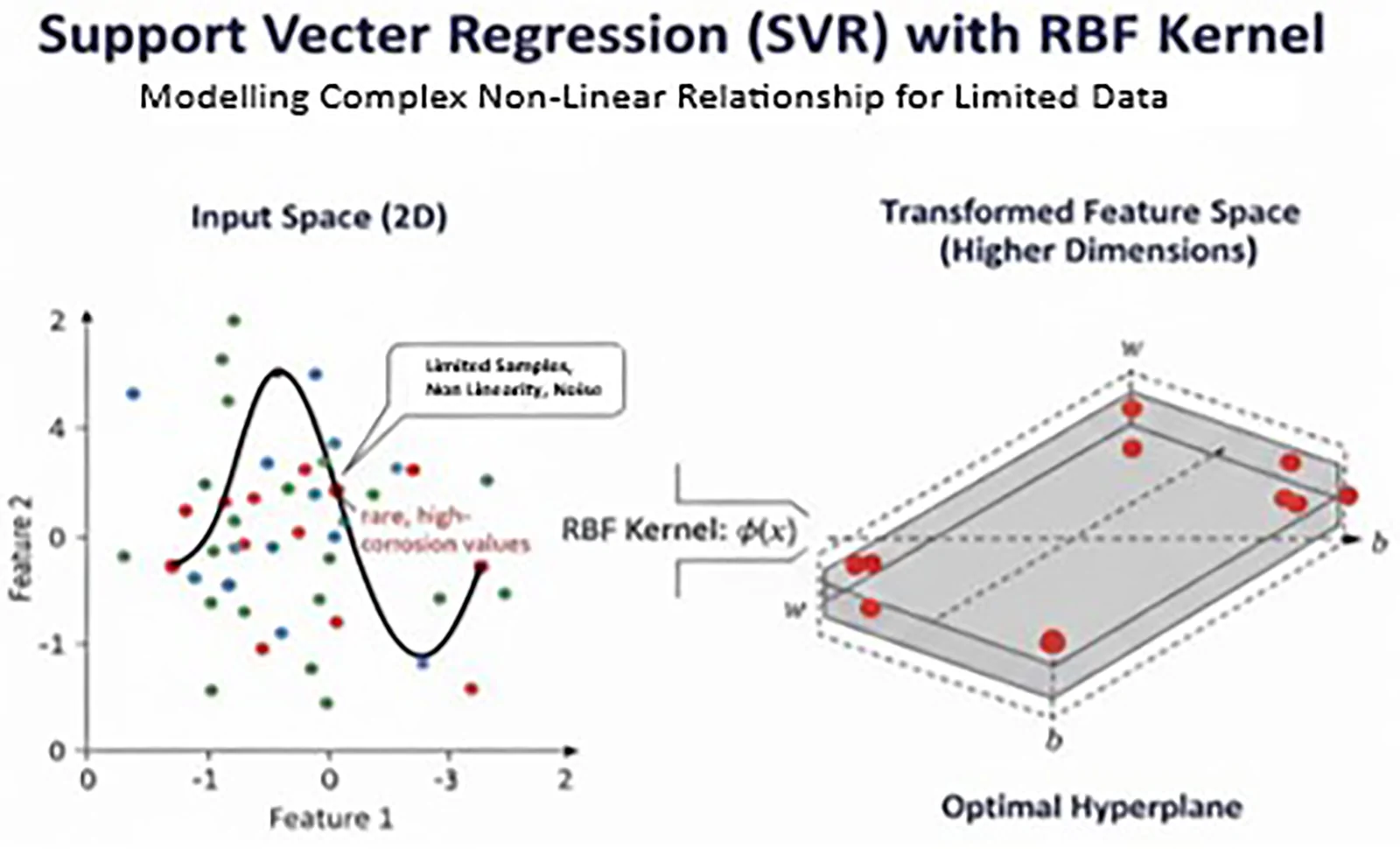
SVR diagram.

Fig 3 shows a general two-dimensional view of the working principle of SVR with a Radial Basis Function (RBF) kernel. When data are plotted in the original input space, the points are usually scattered in a nonlinear fashion, which makes it very hard to find a line or something similar that can represent the data well. However, with the help of the RBF kernel, the points are implicitly changed or “mapped” to a higher-dimensional space, where it is easier to separate things with a line, or in this case, a hyperplane. Therefore, with this change of the space, SVR is able to model very complicated nonlinear relationships in a very effective way, even if only a small and noisy set of data is available.

The mathematical expression of the SVR prediction rule is:


$$\begin{array}{c} \begin{array}{c} f(x_{i})=\langle 𝐰,\phi (x_{i})\rangle +b \end{array}\; \end{array}$$ (2)

where (𝐰) is the weight vector in the transformed feature space, $\begin{array}{c} \begin{array}{c} \left(\phi (x_{i})\right) \end{array}\; \end{array}$ represents the nonlinear mapping of the input features, and (**b**) is the bias term. The inner product $\begin{array}{c} \begin{array}{c} \left(\langle \cdot ,\cdot \rangle \right) \end{array}\; \end{array}$ is computed in the high-dimensional feature space induced by the kernel function.

Nevertheless, Equation (2) merely denotes the regression function.

The entire formulation of SVR is specified through an optimization problem based on the ε-insensitive loss function that permits prediction errors to be within a margin (epsilon) and penalizes only those errors that exceed the margin.

The formulation of the SVR optimization problem is as follows:


$$\begin{array}{c} \underset{𝐰,b,\xi_{i},\xi_{i}^{*}}{min}\frac{\text{1}}{\text{2}}\|𝐰{\|}^{\text{2}}+C\sum_{i=\text{1}}^{n}\left(\xi_{i}+\xi_{i}^{*}\right)\; \end{array}$$ (3)

Subject to:


$$\begin{array}{c} y_{i}-f\left(x_{i}\right)\leq \epsilon +\xi_{i}\; \end{array}$$ (4)

$$\begin{array}{c} f\left(x_{i}\right)-y_{i}\leq \epsilon +\xi_{i}^{*} \end{array}$$ (5)

$$\begin{array}{c} \xi_{i},\;\xi_{i}^{*}\geq \text{0},\; \end{array}$$ (6)

$$\begin{array}{c} i=\text{1},\text{2},\ldots ,n \end{array}$$

Where $\begin{array}{c} \begin{array}{c} \left(\epsilon \right) \end{array}\; \end{array}$ defines the width of the ε-insensitive tube, $\begin{array}{c} \begin{array}{c} \left(\xi_{i}\right) \end{array}\; \end{array}$ and $\begin{array}{c} \begin{array}{c} \left(\xi_{i}^{*}\right) \end{array} \end{array}$ are slack variables representing deviations outside this margin, and (*C*) is the regularization parameter controlling the trade-off between model flatness and tolerance to errors.

The ε-insensitive loss function is expressed as:


$$\begin{array}{c} L_{\epsilon }\left(y,f(x)\right)=\;\left\{ \begin{array}{c} @c\text{0},\;\text{if }\left|y-f(x)\right|\leq \epsilon \;\\ \left|y-f(x)\right|-\epsilon ,\;\text{otherwise}\; \end{array}\;\right. \end{array}$$ (7)

For practical implementation, the dual formulation is used, leading to the regression function:


$$\begin{array}{c} f(x)=\sum_{i=\text{1}}^{n}\left(\alpha_{i}-\alpha_{i}^{*}\right)K\left(x_{i},x\right)+b\; \end{array}$$ (8)

where $\begin{array}{c} \begin{array}{c} \left(\alpha_{i}\right) \end{array} \end{array}$ and $\begin{array}{c} \begin{array}{c} \left(\alpha_{i}^{*}\right) \end{array}\; \end{array}$ are Lagrange multipliers and $\begin{array}{c} \begin{array}{c} \left(K(x_{i},x)\right) \end{array} \end{array}$ is the kernel function, while *b* is the bias (intercept) term of the SVR model. In this study, the RBF kernel is defined as:


$$\begin{array}{c} \begin{array}{c} K(x_{i},x_{j})=\text{exp}(-\gamma ||x_{i}-x_{j}|{|}^{\text{2}})\; \end{array} \end{array}$$ (9)

where $\begin{array}{c} \begin{array}{c} \left(\gamma \right) \end{array}\; \end{array}$ controls the influence of individual training samples in the feature space.

The hyperparameters were tuned to achieve optimal performance, with (C = 5–20), ($\begin{array}{c} \begin{array}{c} \epsilon \end{array} \end{array}$ = 0.01-0-09, and $\begin{array}{c} \begin{array}{c} \left(\gamma =\text{scale}\right) \end{array} \end{array}$. To further address data imbalance, the Synthetic Minority Over-sampling Technique (SMOTE) was applied with a proportion of 22%, resulting in the SVR-SMOTE variant. Overall, SVR provides a theoretically grounded and flexible framework for modeling nonlinear corrosion behavior. Its ability to handle noise, limited data, and complex feature interactions makes it particularly suitable for corrosion depth estimation in buried pipeline systems.

### 3.3. XGboost

Extreme Gradient Boosting (XGBoost) is one of the most popular tree-based ensemble learning methods. It has been utilized in a wide range of environmental and engineering prediction problems thanks to its excellent predictive power and flexibility in handling complex nonlinear relationships. In fact, XGBoost is the most appropriate learning algorithm when the data exhibits feature interaction, noise, or even missing values. Pipelines are susceptible to corrosion, and the progression of corrosion results from nonlinear interactions of soil parameters, such as moisture level, resistivity, and chloride concentration. These variables might cause a sudden or enhanced corrosion effect. XGBoost works by constructing an ensemble of decision trees, which makes it capable of capturing these nonlinearities and interaction effects very well. Researchers have also found it very useful for corrosion prediction, especially when the dataset is complex [54].

In this study, XGBoost is employed as a benchmark ensemble model to evaluate its capability in modeling corrosion depth in comparison with other machine learning and deep learning approaches. The predicted corrosion response is expressed as:


y^i=∑k=1Kfk(xi)                    (10)

where (y^i)  represents the predicted corrosion value for the $\begin{array}{c} \begin{array}{c} \left(i^{\text{th}}\right) \end{array}\; \end{array}$ sample, $\begin{array}{c} \begin{array}{c} \left(x_{i}\right) \end{array}\; \end{array}$ is the corresponding input feature vector, $\begin{array}{c} \begin{array}{c} \left(f_{k}(\cdot )\right) \end{array}\; \end{array}$ denotes an individual regression tree, and (k) is the total number of trees in the ensemble.

During training, XGBoost minimizes a regularized objective function that balances model accuracy and complexity, thereby reducing the risk of overfitting [60]. The objective function combines a loss term, which measures the difference between predicted and observed values, and a regularization term that penalizes overly complex trees. Furthermore, to address data imbalance and improve the model’s sensitivity to under-represented high-corrosion instances, the Synthetic Minority Over-sampling Technique (SMOTE) was integrated into the training process. This hybrid approach (XGBoost-SMOTE) may enhance the model’s ability to learn critical corrosion patterns that may otherwise be overlooked. Overall, XGBoost provides a flexible and well-regularized framework for modeling corrosion behavior, making it a suitable choice for capturing complex relationships in pipeline corrosion data without relying on overly strong assumptions.

### 3.4. MLR

Multiple Linear Regression (MLR) is still one of the major methods for corrosion prediction and is often used as a baseline or a reference model. Despite the fact that corrosion is fundamentally a nonlinear phenomenon, linear models remain extensively used in soil-pipeline research mainly because they allow easy interpretation and, more importantly, they can clearly show the relative impact of each soil parameter [52,53].

Besides, MLR is also very valuable as a point of comparison when judging how well-advanced machine learning (ML) and deep learning techniques perform. It helps establish the level of performance that any new method should at least meet and is therefore an excellent tool when one wants to demonstrate the benefits of synthetic oversampling techniques.

It is important to note that MLR relies on several key assumptions, including linearity between predictors and response, independence of errors, homoscedasticity (constant variance of residuals), normality of residuals, and absence of multicollinearity among predictors. These assumptions could be verified prior to model application using standard diagnostic techniques, including residual analysis, normal probability (Q–Q) plots, and Variance Inflation Factor (VIF) measures.

The relationship between corrosion depth and soil parameters in the MLR framework is expressed as:


$$\begin{array}{c} d_{max,\;i\;}=\;\beta_{\text{0}}+\;\beta_{\text{1}}{S_{\text{1}}}_{i}+\;\beta_{\text{2}}{S_{\text{2}}}_{i}+\;\beta_{\text{3}}{S_{\text{3}}}_{i}+\ldots \beta_{k}{S_{k}}_{i}+\varepsilon_{i}\; \end{array}$$ (11)

Where each term represents:

- $\begin{array}{c} d_{max,\;i\;}\; \end{array}$ = predicted corrosion depth at observation *i*
- $\beta_{\text{0}}$= intercept
- $\beta_{\text{1}}$ **…** $\beta_{\text{1}}$ = regression coefficients for independent variables.
- ${S_{\text{1}}}_{i}$ **…** ${S_{k}}_{i}$= independent variables.
- $\varepsilon_{i}$ = random error term.

### 3.5. SMOTE

Field-collected‍‍‍ datasets often underrepresent severe corrosion depths, which, in turn, results in biased models that have low performance in the critical ranges. SMOTER solves this problem by creating synthetic samples in the regions of the target variable where there are very few observations. The SMOTER algorithm generates synthetic minority-range samples using the following interpolation-based expression (for target values located in minority/rare regions):


$$\begin{array}{c} {\text{Synthetic}}_{i}=\;X_{n}+r.\left(X_{n}-\;X_{m}\right)\; \end{array}$$ (12)

Where:

- $X_{n}$ = feature vector of a minority-region instance
- $X_{m}$ = feature vector of one of its nearest neighbors (within the same target range)
- r = random number in the interval (0, 1) controlling interpolation

The difficulty of modeling corrosion because of its randomness, lack of critical samples, and heterogeneity of the underground environment is acknowledged by the literature [53,56]. The direct introduction of SMOTER into addressing these issues by enriching high-corrosion samples and producing a more balanced data distribution. This paper is among the first to employ SMOTER in the prediction of underground pipeline ‍‍‍corrosion.

It is important to note that the classical SMOTE method is primary used for coping with classification problems, however, it was adapted to deal with regression engineering problems using a polynomial-based SMOTER approach executed via the smote_variants library in Python. In the classification data, the minority classes are explicitly defined, while in regression tasks, the minority regions in the corrosion data are associated with the experimental data points that are under-represented regions of the continuous target variable. Within the framework of predicting corrosion depth, the minority data of *d*_*max*_ represent extreme or rare values, especially corrosion depths that occur less frequently within the dataset but are highly significant for integrity assessment purposes. The adopted polynomial-based SMOTER technique in this paper was used to identify underrepresented regions based on low-frequency areas in the target (*d*_*max*_) distribution. After that, the algorithm generates additional synthetic samples by interpolating between nearby observations to ensure that the relationship between the input features and the continuous output is maintained. Notably, we applied the current framework in order to improve the presence of less frequent corrosion depth values while keeping the data distribution smooth and consistent.

### 3.6. Model development

**Fig 4:**
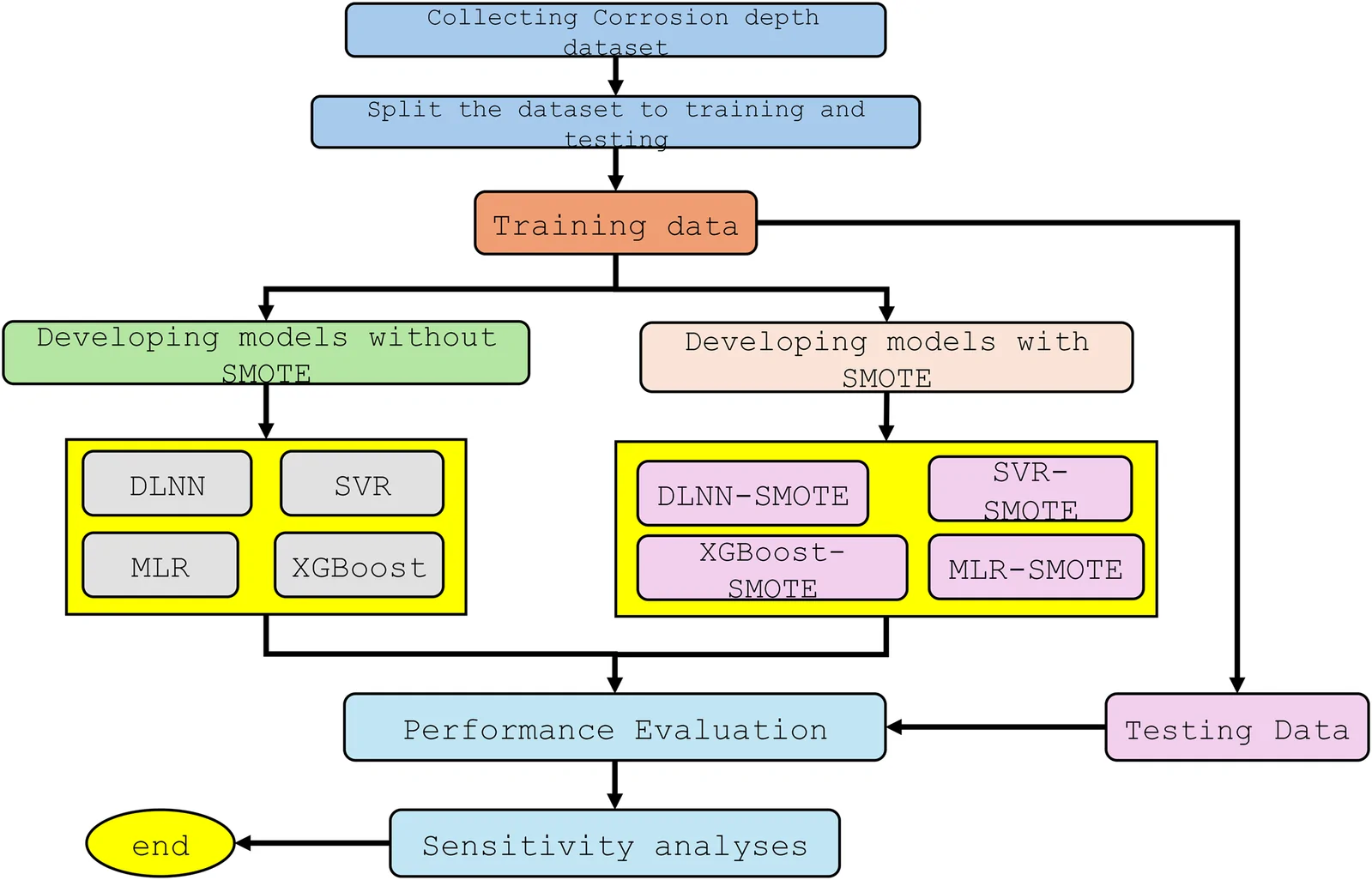
Flowchart of the model development procedure adopted in this study.

In this study, classical ML-based models, including MLR, SVR, and XGBoost, along with a DLNN model, were developed to predict corrosion depth in pipelines (d_max_). The experimental dataset consisted of 259 samples, which were divided into 75% for training and 25% for testing using the train_test_split function from the scikit-learn library in Python. To ensure a fair assessment and reduce any bias from how the samples are arranged, the dataset was randomly split into separate training and testing sets, with the test portion kept completely unseen during model development. Fig 4 illustrates the main steps involved in the development of the adopted modeling framework. Prior to model training, all input features were normalized between 0 and 1 to ensure computational stability and improve learning performance. In this study, SMOTER was applied only to the normalized training data, ensuring that the testing set remained completely unseen during the resampling process [59,60]. The resulting balanced dataset was then used for model training, which helped the models better capture underrepresented corrosion conditions and improved their predictive capability, especially for extreme values.

After tuning the model-specific hyperparameters to their optimal values, performance was evaluated using several statistical metrics. To address data imbalance and examine its impact on predictive accuracy, the SMOTE technique (polynomial variant) was applied to the training data to generate synthetic samples, while the testing set remained unchanged. Each model was then retrained using the augmented dataset and the previously optimized parameters to assess the effect of SMOTE on model behavior. Finally, model performance was further examined through uncertainty and sensitivity analyses to identify the most efficient and reliable prediction model.

### 3.7. Statistical parameters

Various statistical indicators were used to test the ability of the designed models and the evaluation parameters commonly used by the previous authors were taken to ensure a consistent and dependable differential analysis. Error based criteria like Root Mean Square Error (RMSE), Mean Absolute Error (*MAE*), Mean Absolute Percentage Error (*MAPE%*) were utilized keeping the measure of uncertainty at a 95% confidence interval (*U₉₅*) [61].

The correlation coefficient (*R*), Willmott index (*WI*) and coefficient of determination (*R²*), also referred to as the fit adequacy satisfactory metrics, were used to enumerate the degree of similarity between predicted and observed corrosion depth values. These statistical parameters allow the evaluation of complex aspects of a model such as the level of accuracy, the degree of consistency, and the ability for prediction. Mathematical formulas for the evaluation indicators are shown below [62]:


$$\begin{array}{c} MAE=\;\frac{\text{1}}{n}\sum_{i=\text{1}}^{n}|y_{i}-\;\;{\hat{y}}_{i}| \end{array}$$ (13)

$$\begin{array}{c} RMSE=\;\sqrt{\frac{\text{1}}{n}\sum_{i=\text{1}}^{n}{(y_{i}-\;\;{\hat{y}}_{i})}^{\text{2}}} \end{array}$$ (14)

$$\begin{array}{c} R=\;\frac{\sum_{i=\text{1}}^{n}\left((y_{i}-\;\;\overset{―}{y})({\hat{y}}_{i}-\;\;\overset{―}{\hat{y}})\right)}{\sqrt{\sum_{i=\text{1}}^{n}{(y_{i}-\;\;\overset{―}{y})}^{\text{2}}\sum_{i=\text{1}}^{n}{({\hat{y}}_{i}-\;\;\overset{―}{\hat{y}})}^{\text{2}}}} \end{array}$$ (15)

$$\begin{array}{c} WI=\;\text{1}-\;\frac{\sum_{i=\text{1}}^{n}{(y_{i}-\;\;{\hat{y}}_{i})}^{\text{2}}}{\sum_{i=\text{1}}^{n}{(|y_{i}-\;\;\overset{―}{y}|+\;\;\;|{\hat{y}}_{i}-\;\;\overset{―}{y}|)}^{\text{2}}} \end{array}$$ (16)

$$\begin{array}{c} R^{\text{2}}=\;\text{1}-\;\frac{\sum_{i=\text{1}}^{n}{(y_{i}-\;\;{\hat{y}}_{i})}^{\text{2}}}{\sum_{i=\text{1}}^{n}{(y_{i}-\;\;\overset{―}{y})}^{\text{2}}} \end{array}$$ (17)

$$\begin{array}{c} U_{\text{9}\text{5}}=\;\text{1}.\text{9}\text{6}\sqrt{{RMSE}^{\text{2}}+{SD}^{\text{2}}} \end{array}$$ (18)

Where, the variables $y_{i}$, and ${\hat{y}}_{i}$ are the observed and predicted corrosion depth. The $\overset{―}{\hat{y}}$, and $\overset{―}{y}$ are the mean of the forecasted and observed inflow, and the n is the total number of observations.

## 4. Results of the models

The current paper investigates the effectiveness of DLNN and other ML models (SVR, XGBoost, and MLR) in predicting the maximum depth of corrosion in pipes (d_max_). In this work, to enhance prediction accuracy and improve model performance, we used synthetic data to increase the minority rates in the dataset by applying the SMOTE technique. This approach generates synthetic minority class samples to address data imbalance, leading to better performance and more reliable predictions. To obtain a fair assessment, the models were first trained without applying the SMOTE technique, and their hyperparameters were tuned accordingly. In the next step, the same model structures and the same optimized hyperparameters were used to train the models with SMOTE-generated synthetic data. This procedure allows for an accurate evaluation of the specific effect of SMOTE on the prediction performance of the models.

### 4.1. Parameter tuning

The hyperparameters of the deep learning and machine learning models were carefully tuned using a robust two-stage strategy combining Random Search and manual trial-and-error method. Random search was initially deployed to broadly scan the high-dimensional parameter space and isolate highly prospective configurations. Subsequently, fine-grained trial-and-error was used to meticulously adjust and check the parameters within narrow, specific ranges to optimize model capacity and ensure peak performance.Per the baseline split architecture established, overfitting was rigorously monitored and controlled by evaluating the structural consistency between the training performance metrics and an independent, completely unseen testing dataset (25%). The objective metrics utilized to select the optimal parameters were the minimization of *RMSE*, *R*, and *MAE* during the training phase, while safeguarding model generalization capacity on the testing data. The adopted process involved adjusting each model-specific setting to improve accuracy and reduce the errors to the lowest level. The final optimized parameters for the applied models are presented in Table 2.

**Table 2:** Final tuned hyperparameter settings for optimal model performance.

Model	Hyperparameters
SVR, and SVR-SMOTE	• Kernel is Radial Basis Function • C = 10.0 • epsilon = 0.05 • gamma = ‘scale’ • proportion of SMOTE = 22
XGBoost, and XGBoost-SMOTE	• Number of trees = 500, • Learning rate = 0.050.07 • Minimum child weight = 1 • Maximum tree depth = 4–6 • Subsample ratio = 0.8 • Column sample per tree = 0.8–0.89 • L1 regularization = 0.63–0.1 • L2 regularization = 0.95–1 • Gamma = 0.000, • proportion of SMOTE = 34
MLR, and MLR-SMOTE	• proportion of SMOTE = 2
DLNN, and DLNN-SMOTE	1. Validation split = 0.2 2. Epochs = 500 3. Batch size = 32 4. Number of neurons in the first hidden layer = 128(100–150) 5. Number of neurons in the second hidden layer = 64 6. Number of neurons in the third hidden layer = 32 7. Number of neurons in the fourth hidden layer = 25 8. Number of neurons in the fifth hidden layer = 20 9. Number of neurons in the sixth hidden layer = 10 10. Number of neurons in the output hidden layer = 1 11. proportion of SMOTE = 34

After tuning the model parameters, all models showed noticeable improvements in performance.

For instance, the SVR model achieved an *R* value of 0.881 and an *RMSE* of 0.984 in the testing phase using the hyperparameter setting of *C* = 10, *epsilon* = 0.05, and gamma set to “*scale*,” i,e. the gamma value is optimised by the algorithm adaptively. On the same note, the SVR-SMOTE model demonstrated an increased minority class by 22%, thereby achieving a lower *RMSE* of 0.766 and a higher correlation of *R* = 0.913.

After parameter tuning, the XGBoost model reached an *RMSE* of 0.897 when tested with 500 estimators, minimum child weight = 1, and subsample ratio = 0.8. However, it was found to be sensitive to the learning rate, and performed best between 0.05 and 0.07, with a maximum tree depth of 4–6, and *L1* regularisation between 0.03 and 0.10. Under these settings, the model produced a strong predictive accuracy of *R* = 0.858. This model, however, required a larger minority population (32%) than SVR to achieve performance gains. After adding synthetic data representing about 32% increase in the minority class, the XGBoost-SMOTE model achieved further improvement, lowering *RMSE* to 0.807. Finally, the DLNN model showed stable performance when configured with six hidden layers, achieving an *RMSE* of 1.088. However, error increased by 20–30% when using hidden layer counts between two and seven. The model required SMOTE population of 32% to improve performance and enhance predictive stability. At this configuration, the DLNN-SMOTE model demonstrated its best generalization capability, achieving *R* = 0.940 and *RMSE* = 0.624.

Accordingly, the above results illustrated that the DLNN is the best model as well as requires more data samples generated by SMOTE approach, which has a population size of 534. At this stage, it is vital to explore whether the synthetic data is logical and physically realistic. Consequently, the generated corroded samples were evaluated using statistical and distributional comparisons of the training population before and after resampling. The quantitative results show that the size of the training population increased from 194 to 534 samples, and such an increase in the training samples confirms effective augmentation of under-represented regions in the corrosion depth space. The other significant observation is that the key statistical parameters like the maximum and minimum corrosion depth remained unchanged (0.14–8.56), which indicates that the applied SMOTE-based algorithm adhered to the physical limits of the experimental data and no extrapolation beyond the physically observed range was introduced. Although variations in the mean (1.84 to 0.952) and standard deviation (std ranges from 1.662 to 1.103) were observed, which improved the representation of sparse regions in the population rather than the distortion of the dataset. In conclusion, it is notably remarked that the generated synthetic samples remain physically realistic and consistent with the underlying distribution of the original population.

### 4.2. Application results

The performance of the applied models in predicting *d*_*max*_ was evaluated using several statistical metrics before and after applying the SMOTE technique. Initially, without SMOTE, the models generally showed good performance during the training phase, as reported in Table 3. XGBoost and DLNN exhibited almost remarkable accuracy, achieving low *RMSE* values of 0.650 and 0.659, respectively, and XGBoost slightly outperformed DLNN with *MAE* = 0.387 and *MAPE* = 28.74%, compared with *MAE* = 0.412 and *MAPE* = 34.50% for DLNN. The SVR model delivered lower prediction accuracy than both DLNN and XGBoost, with higher error values (*MAE* = 0.514, *RMSE* = 0.568, *MAPE* = 43.52%), while the MLR model provided the weakest performance, showing the highest prediction errors. After implementing SMOTE, all AI models demonstrated significant improvements in the training phase. The DLNN-SMOTE model achieved the best performance, with *RMSE* = 0.489, *MAE* = 0.380, *MAPE* = 23.98%, *d* = 0.985, and *R* = 0.986, while XGBoost-SMOTE and SVR-SMOTE also yielded enhanced accuracy, producing *RMSE* = 0.513/ 0.620, *MAE* = 0.397/ 0.520, *R* = 0.981/ 0.959, and *d* = 0.983/ 0.976, respectively. Overall, the integration of synthetic data using SMOTE improved the learning capability of all models, resulting in a marked reduction in prediction error metrics.

**Table 3:** Performance metrics for the training phase.

	Models	*RMSE*	*MAE*	*MAPE %*	*d*	*R*
Before applying SMOTE	DLNN	0.659	0.412	34.50	0.974	0.951
	SVR	0.568	0.514	43.52	0.981	0.965
	XGBoost	0.650	0.387	28.74	0.972	0.959
	MLR	1.701	1.056	73.79	0.714	0.605
After applying SMOTE	DLNN-SMOTE	0.489	0.380	23.98	0.985	0.986
	SVR-SMOTE	0.620	0.520	41.56	0.976	0.959
	XGBoost-SMOTE	0.513	0.397	24.96	0.983	0.981
	MLR-SMOTE	1.708	1.048	72.74	0.690	0.604

The outcomes of the models’ performances in the testing phase, before and after generating synthetic data using SMOTE, are evaluated and summarized in Table 4. The results show that all models exhibited relatively higher prediction errors prior to applying SMOTE. After introducing synthetic data, a substantial reduction in prediction error was observed for all AI models, with DLNN showing the most significant improvement, achieving *RMSE* = 0.624, *MAE* = 0.490, and *R* = 0.940. These results indicate that statistical metrics such as *RMSE*, *MAE*, *MAPE*, *d*, and *R* improved by 42.65%, 31.28%, 37.43%, 6.86%, and 13.94%, respectively, due to applying SMOTE. Notably, the MLR model remained the weakest performer both before and after SMOTE compared with the AI-based models. The second- and third-best models were SVR-SMOTE and XGBoost-SMOTE, which produced *RMSE* = 0.766/ 0.807, *MAE* = 0.577/ 0.584, and *R* = 0.913/ 0.873, respectively. Overall, all AI models benefited from the application of SMOTE, showing substantial reductions in testing-phase error metrics, with *RMSE*, *MAPE* and *MAE* decreasing by 24.94%, 24.23% and 18.60%, respectively.

**Table 4:** Performance metrics for testing phase.

	Models	*RMSE*	*MAE*	*MAPE %*	*d*	*R*
Before applying SMOTE	DLNN	1.088	0.713	47.98	0.904	0.825
	SVR	0.984	0.714	54.49	0.929	0.881
	XGBoost	0.897	0.617	40.99	0.923	0.858
	MLR	1.179	0.774	52.45	0.838	0.738
After applying SMOTE	DLNN-SMOTE	0.624	0.490	30.02	0.966	0.940
	SVR-SMOTE	0.766	0.577	42.78	0.953	0.913
	XGBoost-SMOTE	0.807	0.584	35.34	0.930	0.873
	MLR-SMOTE	1.173	0.736	47.18	0.821	0.740

**Fig 5:**
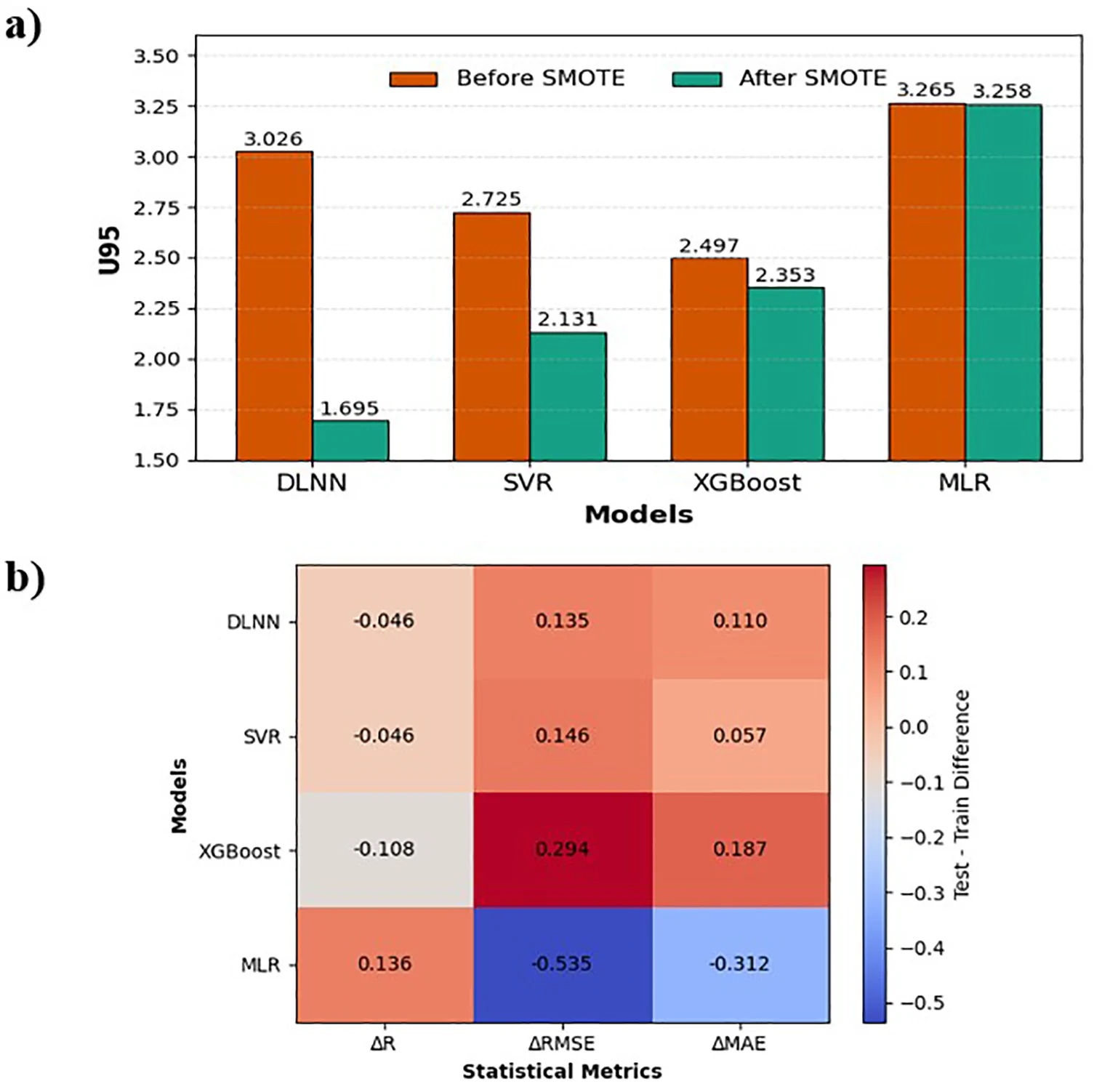
Evaluation of the performance of the models used. a) Model performance comparison using U95 before and after SMOTE application. b) Generalization gap to evaluate the models supported by SMOTE generalization capacity.

Fig 5a compares the models’ performances before and after applying SMOTE using the uncertainty metric (*U*_*95*_) over testing dataset. Such visualizations provide a clear and fair comparison for evaluating how the SMOTE approach affects the adopted models. Most of the models show notable improvement after applying SMOTE, with DLNN demonstrating the greatest enhancement due to the inclusion of synthetic data, whereas MLR records the highest *U*_*95*_ value after SMOTE. Overall, the application of SMOTE positively influences the performance of the models and enhances their predictive capabilities. The generalization performance of the applied models supported by SMOTE were compared according to their performance in training and testing steps. Based on the reported results of the key statistical parameters as shown in Fig 5b, the differences between training and testing remain small across all models, which suggests that the models are behaving consistently on new data. The findings showed that some models such as DLNN-SMOTE, and SVR-SMOTE show the closest agreement between the two datasets, while XGBoost-SMOTE has a slightly larger shift in performance. The MLR-SMOTE model, on the other hand, performs less strongly overall but does not show signs of overfitting. Overall, the patterns across the metrics indicate stable generalization under the random split setup, with no clear evidence that the models are overfitted.

**Fig 6:**
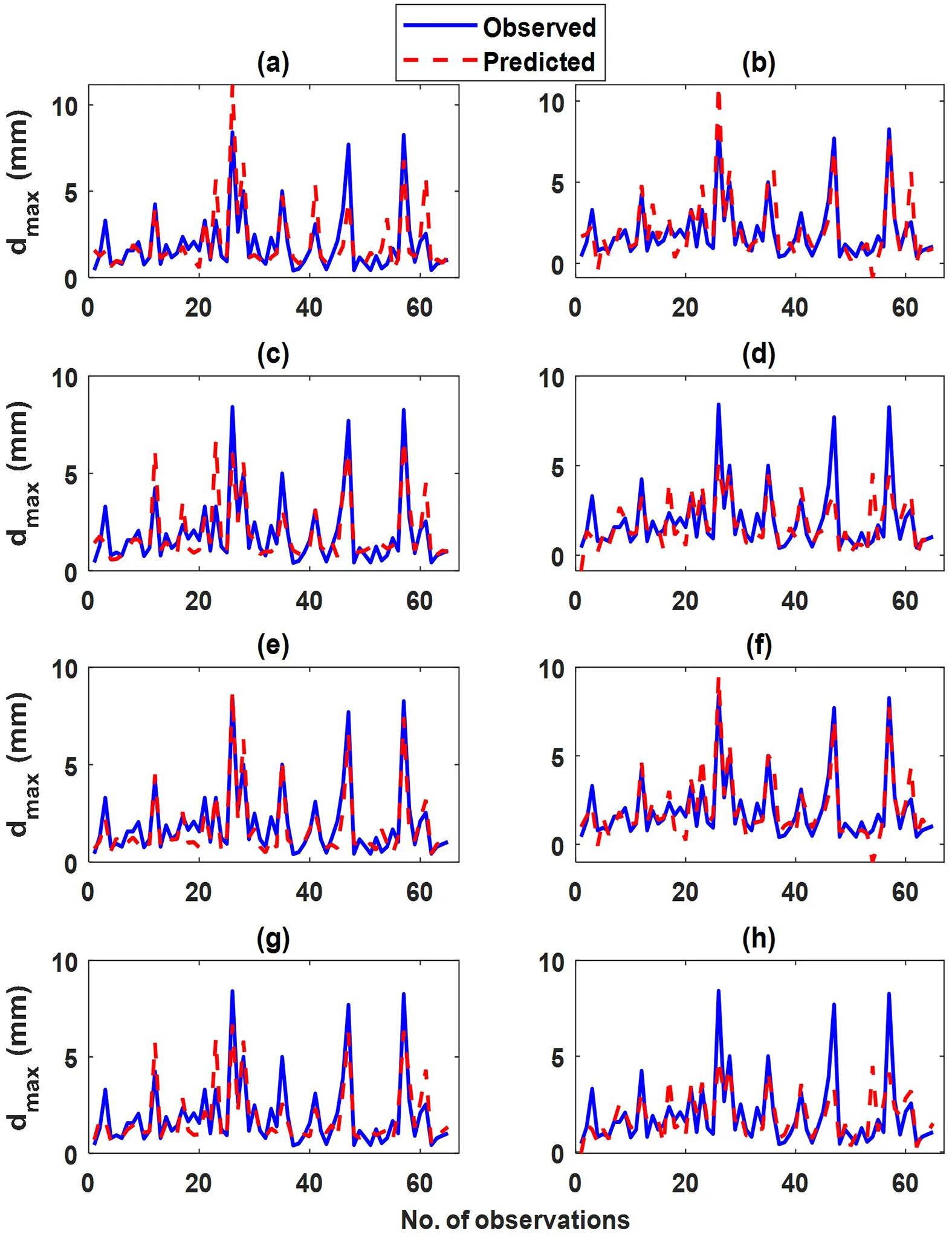
Comparison of observed and predicted values for different regression models: (a) DLNN, (b) SVR, (c) XGBoost, (d) MLR, (e) DLNN-SMOTE, (f) SVR-SMOTE, (g) XGBoost-SMOTE, and (h) MLR-SMOTE.

**Fig 7:**
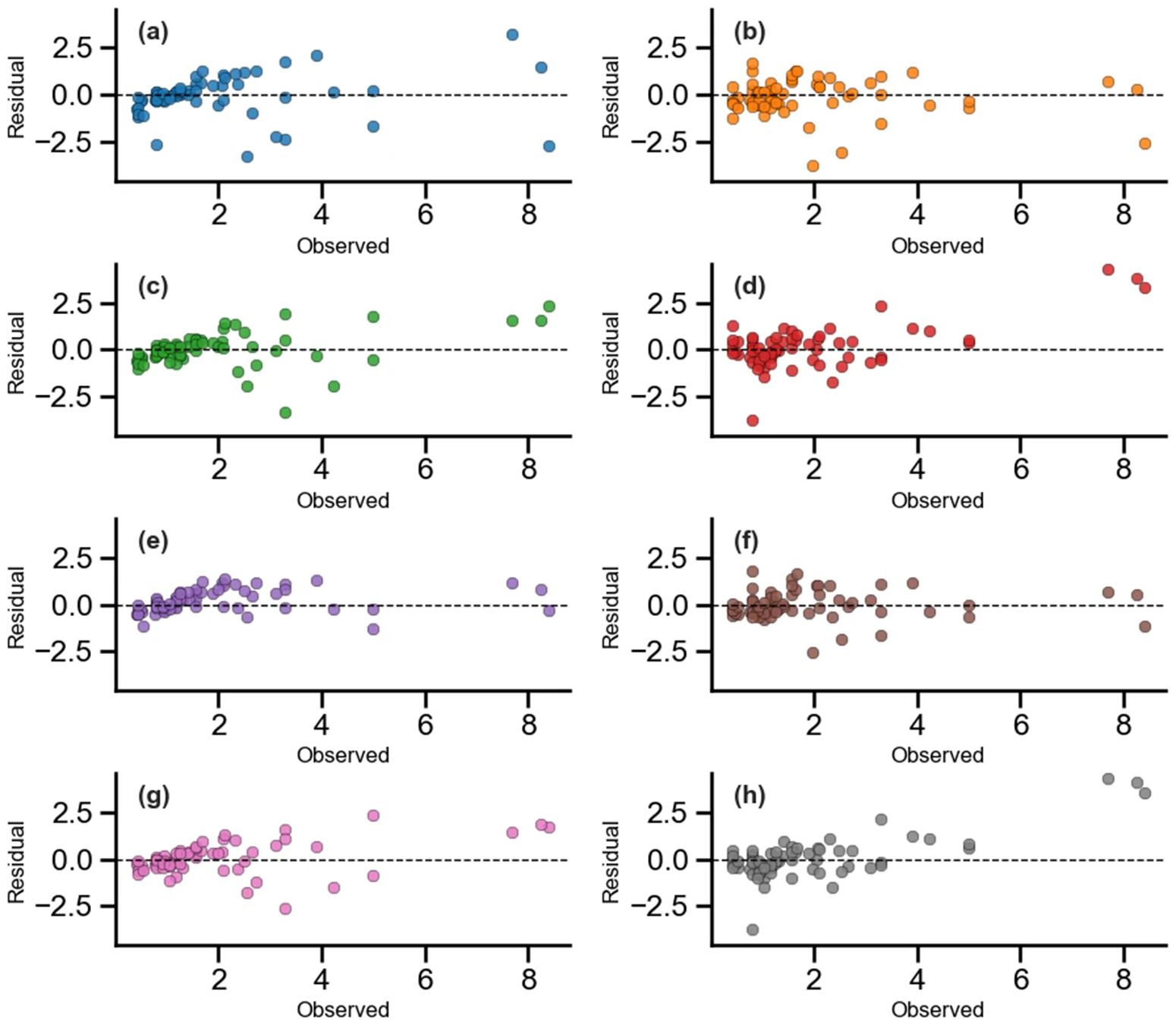
Residual plots showing the difference between observed and predicted maximum corrosion depth values for all developed models. Subplots (a)–(h) correspond respectively to DLNN, SVR, XGBoost, MLR, DLNN-SMOTE, SVR-SMOTE, XGBoost-SMOTE, and MLR-SMOTE models.

The line graph presented in Fig 6 provides a direct comparison between the observed and predicted values. The figure shows that the outputs of the DLNN-SMOTE and SVR-SMOTE models exhibit strong alignment with the observed data points, with a noticeably better performance for the DLNN-SMOTE model in capturing the overall data pattern, including peak, maximum, and minimum values. In contrast, the AI models without SMOTE show several predicted points that fall at relatively larger distances from the observed values compared with the SMOTE-supported models. The residual errors of each predicted point in Fig 7 against the observed records help identify where the models slightly overestimate or underestimate the true values. As observed, the DLNN-SMOTE model residuals that lie closer to the zero line, with no trace of a trend in error distribution, hence establishing strong predictive capability and stability of the model.

**Fig 8:**
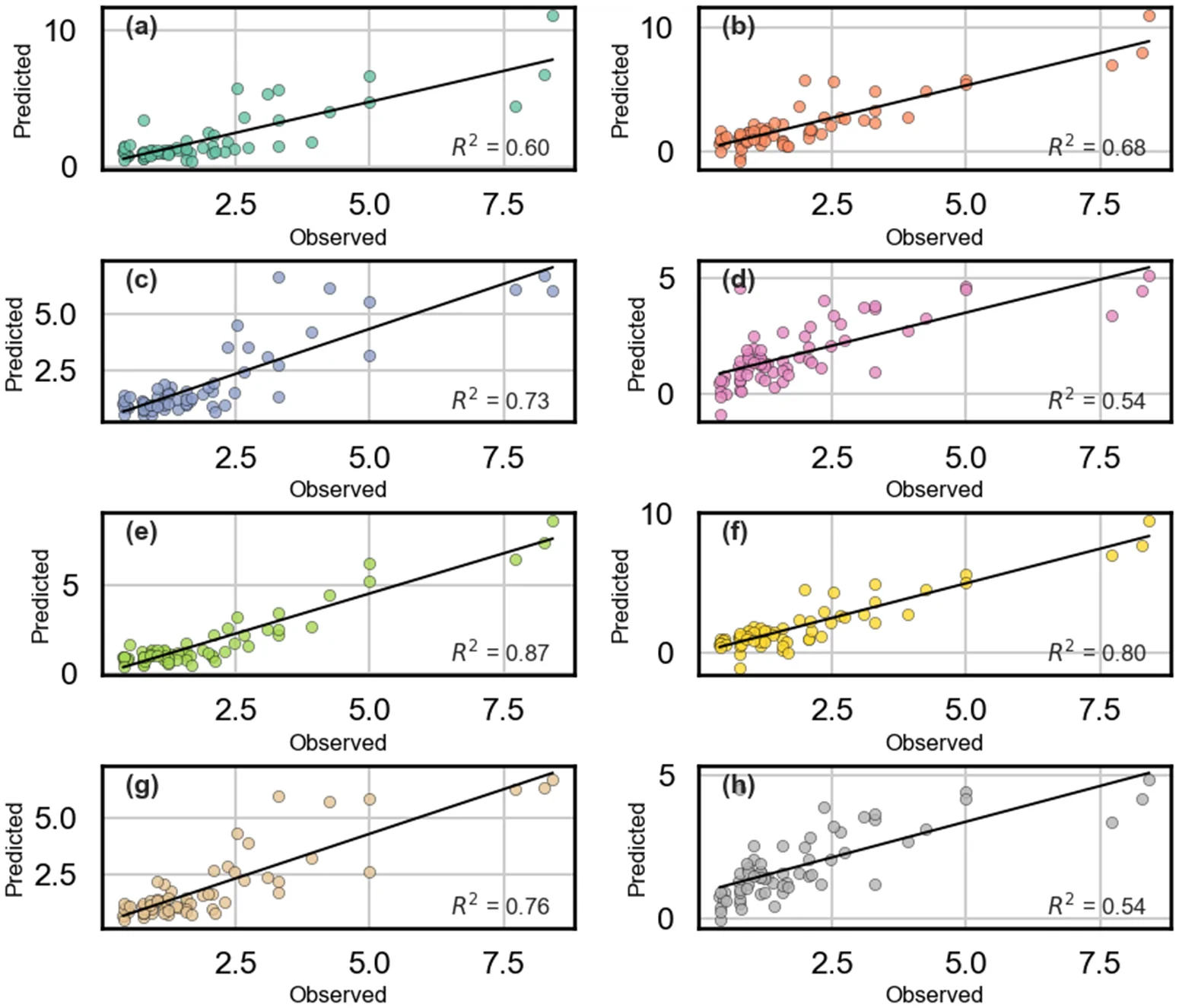
Comparison between observed and predicted *d*_*max*_ values obtained from different models. Subplots (a)–(h) correspond respectively to DLNN, SVR, XGBoost, MLR, DLNN-SMOTE, SVR-SMOTE, XGBoost-SMOTE, and MLR-SMOTE models.

Fig 8(a–h) depicts the linear relationship between the observed and predicted values, and helps visualize the position of each predicted point, providing information on the deviations of the predictions from the observed values, as well as presenting the corresponding *R²* values. The figure highlights the DLNN-SMOTE model as the best-performing prediction model, producing more accurate results with *R²* = 0.87. Where other models exhibit varying accuracy levels with *R²* values ranging from 54% to 80%, DLNN-SMOTE explains 87% of the variability in the measured corrosion dataset.

**Fig 9:**
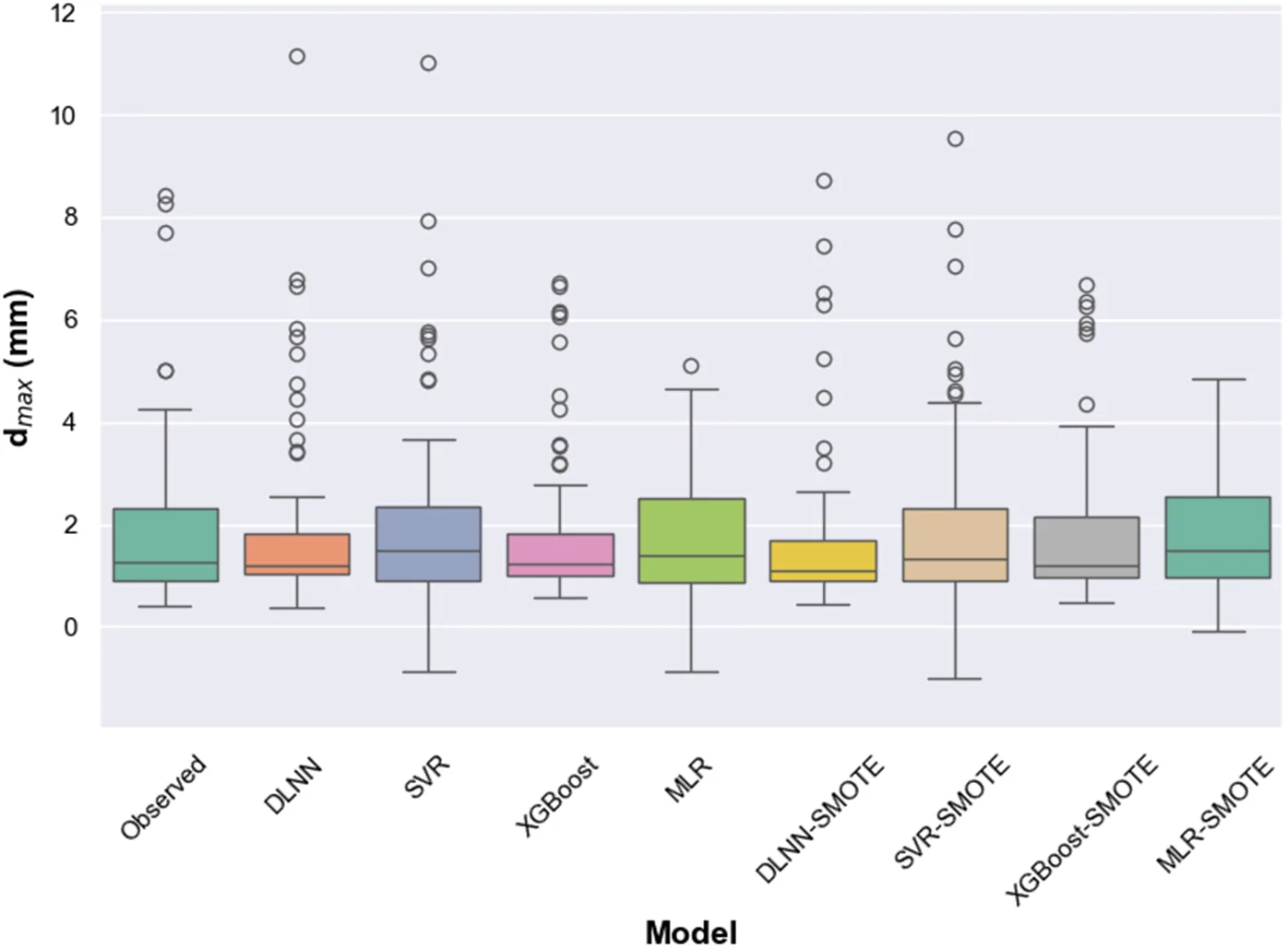
Boxplot depicting the performance scores of various predictive models versus observed values.

Fig 9 displays corrosion values with the prediction accuracy of the developed models before and after implementing SMOTE. The box plot shows median values by the horizontal line inside each box, with DLNN-SMOTE median value very close to the observed data, indicating that the model effectively captures both the central tendency and variability of corrosion depth. Observation to be remarked was the increased performative ability of other models after applying SMOTE, while earlier they were found struggling to reproduce the highest values and produced larger errors. The figure entails that the SVR-based model and the MLR model’s overall performance and handling of extreme values improved but they were still generating some negative values. To conclude, the boxplots establish that hybrid deep learning models outperform traditional models in preserving the statistical characteristics of the observed corrosion values.

**Fig 10:**
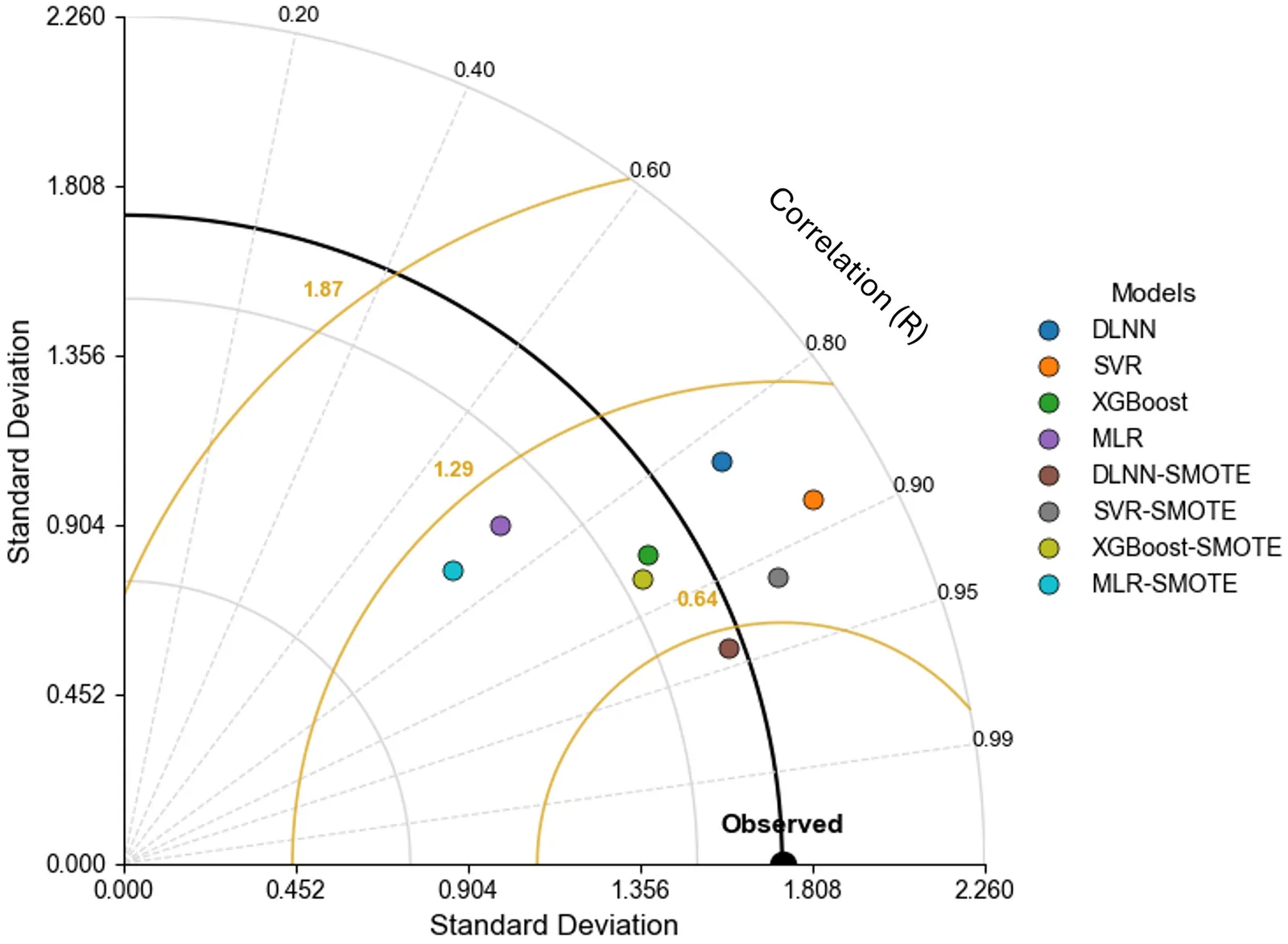
Taylor diagram illustrates the performance of ML and DL models before and after SMOTE.

Fig 10 presents a concise graphical evaluation of model performance by integrating three statistical metrics: correlation coefficient (R), standard deviation, and centered RMSE. In this diagram, models positioned closer to the reference point (Observed) indicate superior overall performance via Taylor diagram. As depicted the DLNN-SMOTE model is demonstrating the highest correlation with the observed corrosion depth located nearest to the reference, and the closest agreement in standard deviation. A small centered RMSE further attests its predictive capability. It can also be viewed that SMOTE approach improves the consistency of the results with the observed values, as reflected by AI models closer proximity to the reference point compared to the models without SMOTE.

Wilcoxon signed-rank test implemented in Python via the SciPy library is utilized to check whether the prediction accuracy of that model compared to others is statistically significant. The test was applied to paired squared prediction errors between the DLNN-SMOTE model and the benchmark models, as this non-parametric method does not assume normality of the data distribution and a significance level of 0.05. Table 5 shows the results of the test, which indicate that all comparisons are statistically significant (p < 0.05), with DLNN-SMOTE showing significant differences against all comparable models.

**Table 5:** Wilcoxon Signed-Rank Test results for comparing DLNN-SMOTE with other models.

Model 1	Model 2	Statistic	p-value	Better Model	Status
DLNN-SMOTE	SVR-SMOTE	12116	9.21 × 10^−05^	DLNN-SMOTE	Significant
DLNN-SMOTE	XGBoost-SMOTE	6385	4.73 × 10^-18^	DLNN-SMOTE	Significant
DLNN-SMOTE	MLR-SMOTE	8018	2.74 × 10^-13^	DLNN-SMOTE	Significant

### 4.3. Sensitivity analysis

This section of the study analyzes the relative importance of each input variable in predicting pipe corrosion depth (d_max_), both before and after applying the SMOTE balancing technique, with full-input model (including all variables), DLNN, as the baseline for comparison, since it demonstrated the best predictive performance compared to other models. Each input variable was removed individually, and the model was retrained under the same conditions and hyperparameters to assess the resulting performance degradation. The observed changes in *R²* and *RMSE* after each variable was excluded. The research denoted, sensitivity analysis provides a direct measure of the contribution of each feature to prediction accuracy and reveals whether the application of SMOTE altered the importance ranking of the variables.

From Table 6 we can interpret that the removal of pH leads to the greatest increase in prediction errors in both scenarios. This means that pH is the most influential parameter, which aligns with findings reported in previous research. Other parameters like c, t, and pp also increase prediction error when removed, out of these cc too plays a crucial and significant role, whereas t and pp, their influence is noticeably reduced after applying the SMOTE technique. The rest parameters, parameters bc, sc, bd, and rp exhibit only marginal effects, indicating comparatively lower sensitivity. The results further reveal that the re parameter has a substantial impact on prediction error when the model is trained without SMOTE, while its removal becomes less influential once SMOTE is applied. However, ranking of variable importance remains largely consistent after SMOTE, suggesting that SMOTE improves the DLNN model’s predictive performance without altering the fundamental relationships between input variables and corrosion behavior.

**Table 6:** Results of the sensitivity analysis based on sequential variable removal.

Excluding SMOTE					Including SMOTE			
Remove Variable	Training		Testing		Training		Testing	
	*R* ^*2*^	*RMSE*	*R* ^*2*^	*RMSE*	*R* ^*2*^	*RMSE*	*R* ^*2*^	*RMSE*
t	0.298	1.790	0.425	1.312	0.769	1.026	0.688	0.967
pH	0.159	1.959	0.337	1.408	0.624	1.311	0.448	1.284
pp	0.886	0.722	0.479	1.248	0.883	0.732	0.648	1.027
re	0.287	1.805	0.503	1.219	0.852	0.823	0.794	0.785
wc	0.885	0.724	0.724	0.908	0.862	0.794	0.740	0.881
bd	0.669	1.230	0.696	0.954	0.949	0.484	0.813	0.747
cc	0.249	1.851	0.468	1.262	0.530	1.464	0.564	1.142
bc	0.719	1.133	0.722	0.912	0.823	0.900	0.699	0.949
sc	0.915	0.624	0.723	0.910	0.874	0.759	0.778	0.816
rp	0.900	0.677	0.641	1.036	0.966	0.392	0.791	0.790
None	0.904	0.659	0.681	1.088	0.972	0.489	0.884	0.624

## 5. Discussion

The research critically reveals noticeable improvement in model performance after applying SMOTE compared with using the original, imbalanced dataset. RMSE dropped by 25% after SMOTE was applied to the AI models indicating that this technique effectively mitigated the data imbalance in the pipeline corrosion dataset. The models were able to the peak (maximum) corrosion depth, as shown in Fig 6, and 9 due to generation of synthetic samples by SMOTE for the minority class, so the extreme pit-depth values, which are originally represented by only a few samples, become more adequately represented during training. Consequently, the models learn the behavior associated with severe corrosion more accurately and are less prone to underestimating these extreme cases, which is a common limitation of conventional approaches. Benefiting from the deep network’s ability to learn complex nonlinear patterns and from the enriched training data the DLNN with SMOTE lead to more accurate prediction of maximum corrosion depth.

The performance of the DLNN model equipped with SMOTE, selected as the best model in this study, should be evaluated against other prediction models and unseen datasets to verify its effectiveness and accuracy. These comparisons provide deeper insight into the performance of the adopted models and help determine whether they are truly superior or require further improvement. In this study DLNN-SMOTE was compared with several ML models, its performance was also examined relative to models developed in previous studies. In a recent study, researchers employed hybrid ML models such as SVR, extreme learning machine (ELM), Multivariate Adaptive Regression Spline (MARS), and XGBoost, integrating them with a Differential Evolution algorithm to enhance their performance. The results showed that the best model was XGBoost, achieving an *R^2^* of 0.8407 [54]. Another recent study applied regression tree–based models such as Random Forest, XGBoost, Categorical Boosting (CatBoost), and Light Gradient Boosting Machine (LightGBM) to predict the maximum depth of corrosion [40]. Results revealed that CatBoost was the most superior model, achieving an *R^2^* of 0.5924 and an *RMSE* of 0. 833. Another notable investigation highlights developing an SVR model enhanced with improved particle swarm optimization and the EMD algorithm with the aim to improve model performance and combat noise in the features [63]. This study enumerated 120 samples, and the proposed hybrid model demonstrated higher predictive accuracy, achieving an *R^2^* of 0.925. atlast, this reseach consistently remarks consistent results of DLNN-SMOTE and is in line with previous research outcomes, with a superior predictive performance (*R^2^* of 0.94).

IoT sensors installed along the pipeline continuously measure variables such as wall thickness, soil moisture, and pH, and transmit these data in real time to a central platform. This offers an effective strategy for managing corrosion in buried pipelines coupled with data-driven prediction. The data from the central platform is to be analyzed by the machine learning models to estimate pitting depth and identify sections of the pipeline that are more likely to experience severe damage to detect corrosion problems at an earlier stage, so the pipeline does not need to be checked on site as often. The results generated by these machine learning models forms the foundational evidence to plan inspection and maintenance based on risk, giving priority to sections with higher predicted damage, thus assisting to reduce maintenance costs, support a longer pipeline service life, and lowering the chance of leaks or failures.

## 6. Conclusion

The current paper investigates the performance of several machine learning models SVR, XGBoost, MLR, and DLNN model both with and without applying a data-imbalance handling technique (SMOTE) to predict the maximum corrosion depth using a localized dataset of 259 samples. The SMOTE technique generates synthetic samples to enhance the model’s learning process, particularly for minority cases. The study evaluates whether the SMOTE approach improves the predictive performance of the ML and DL models. The results show that the SMOTE-based models achieve significantly better performance than their standalone counterparts, with notable improvements in *MAPE*, *U*_*95*_, *RMSE*, and *R^2^*. In particular, all SMOTE-based models exhibit approximately a 25% reduction in RMSE. Among them, the DLNN-SMOTE model performs the best, achieving an *RMSE* of 0.624, *MAE* of 0.490, *MAPE* of 35.34%, and an *R²* of 0.940. To capture peak corrosion depths with strengthened capability to represent the complex dynamics of corrosion processes, it’s advisable to integrate predictive models with SMOTE, as observed during the sensitivity analysis DLNN’s predictive performance enhanced after applying SMOTE even when critical parameters are missing. The analysis also shows that pH and cc are crucial parameters that play a significant role in predicting corrosion depth.

## 7. Limitation and future work

This study does not include other regression imbalance techniques such as SMOGN, which could provide additional insight into performance comparison. This is due to the focus on a unified SMOTE-based resampling setup with polynomial interpolation. In future work, we encourage researchers to include and evaluate such methods to further validate the proposed approach.

## Supporting information

S1 File Codes and dataset. (ZIP)
