# Supplementary material for: Improving corrosion depth estimation in buried pipelines using SMOTE-integrated machine and deep learning models
Source: PLoS One. 2026 Aug 27;21(8):e0355187. doi: 10.1371/journal.pone.0355187 (PMC13521352; doi:10.1371/journal.pone.0355187)
Supplement: Supplementary file 1 [file pone.0355187.s003.docx]

**Dear Editor,**

We sincerely thank you for the opportunity to submit the revised version of our manuscript to your respected journal. The authors would also like to express their gratitude to the reviewers for their valuable time and constructive comments, which have greatly helped improve the quality of the paper. We confirm that we have made our best efforts to address all reviewer comments and improve the manuscript accordingly. In addition, the authors have clearly provided and clarified the data source and codes used in this study.

Below is a point-by-point response to the reviewers’ comments

**Reviewer #1:** This paper investigates the use of SMOTE (Synthetic Minority Over‑sampling Technique) to address data imbalance in predicting maximum corrosion depth of buried pipelines. It compares four models (DLNN, XGBoost, SVR, MLR) on original vs. SMOTE‑augmented datasets and reports that DLNN‑SMOTE achieves the best performance (RMSE= 0.624, R= 0.940). Here are several suggestions for you to revise the manuscript.

1. How did you adapt SMOTE for the continuous target variable (corrosion depth), and what exactly defines a “minority” region in your regression setting?

**Response:** *Thank you for this comment. In this study, SMOTE was adapted for regression using a polynomial-based SMOTER approach. In regression, the “minority” is not a class but corresponds to under-represented regions of the continuous target variable. In our case, the extreme corrosion depth values are relatively rare and were treated as minority regions based on their low frequency in the target distribution. SMOTE generates synthetic samples in these regions through interpolation between neighboring data points, thereby improving their representation and enhancing model learning. More details have been added to the revised manuscript* ***(Kindly, see pages 20, and 21).***

2. How do you validate that the synthetic samples generated by SMOTE are physically realistic and do not cause overfitting, given your small real dataset (259 samples)?

**Response:** *Thank you for your valuable input*

a) *We have evaluated the physical realism and statistical validity of the generated synthetic samples using a residual- and distribution-based analysis. The results show that key statistical bounds (minimum and maximum corrosion depth) remain unchanged, indicating that no unrealistic values or extrapolation beyond the physical limits of the original dataset were introduced, while only the distributional characteristics (mean and standard deviation) vary slightly due to improved representation of under-represented regions. For more information,* ***please see Page 27****, where it has been clarified that the synthetic samples generated remain physically realistic and consistent with the underlying data distribution.*
b) *We appreciate this important concern regarding the relatively small dataset size (259 samples). To address the risk of overfitting, the data were randomly divided into separate training and testing sets, ensuring that model evaluation was carried out on unseen data (****please see Page 21, section 3.6)****.*
c) *Furthermore, the Model behavior was then assessed by examining the consistency between training and testing performance. The observed results show stable and comparable behavior across both phases, indicating good generalization ability.* ***Please see Page 31 and Figure 5b*** *for more detailed information.*

3. Why did you not include statistical significance tests (e.g., t-test) or other imbalance methods (e.g., SMOGN, weighted loss) as baselines for comparison?

**Response*:*** *We thank the reviewer for this valuable suggestion.*

i. *To check whether the performance differences between competing models are statistically significant, we applied the Wilcoxon signed-rank test, as it is a non-parametric method that does not assume normality of the performance distributions. The results confirm that the improvements of the proposed method over baseline models are statistically significant.* ***Please find more information on page 39.***
ii. *In this regression study, we use SMOTE with polynomial interpolation to generate synthetic samples to address data imbalance. The experiments are conducted within this resampling-based framework and compared against standard regression baselines under the same setting. A limitation of this study is that it does not include other imbalance-aware regression methods such as SMOGN, which could further strengthen the evaluation, and we have highlighted this in the limitations section of the revised manuscript (****Please see Page 43****). This will be addressed in future work by incorporating and comparing such approaches.*

Reviewer #2: Comments:

• To the best of the reviewer's knowledge, optical sensing technology for corrosion depth detection is a highly promising technique that can also serve as a reliable data source for machine learning and deep learning. However, the introduction to this field within the paper remains somewhat insufficient. The authors are advised to refer the following references: https://www.mdpi.com/1424-8220/26/9/2641, https://www.sciencedirect.com/science/article/abs/pii/S0030399224000112, <https://www.sciencedirect.com/science/article/abs/pii/S0263224125018780>.

**Response:** *Thank you for your valuable suggestion. We have revised the introduction to include optical sensing technologies for corrosion depth detection, and we have incorporated the recommended references to highlight their relevance for providing reliable non-destructive data for machine learning and deep learning models.* ***Please see pages 3 and 4*** *for more information.*

• In Section 2, it is stated that this dataset is derived from "public literature" and a previous technical note [47]; however, the process by which the data was compiled is not explicitly described. Please clarify the following: How were these 259 data points assembled (e.g., were they drawn from multiple studies or a single source)? What criteria were used for the inclusion or exclusion of data? Were any preprocessing steps performed (e.g., normalization, outlier removal, handling of missing data)?

**Response:** *The authors thank the reviewer for this comment. The dataset used in this study was adopted directly from previous work, where it was originally compiled from field observations across multiple pipeline excavation sites. No additional data sources were incorporated, and no inclusion or exclusion criteria were applied. The dataset contains no missing values, and all available records were retained. Prior to model development, the data were normalized to ensure consistent feature scaling, and no outlier removal was performed. These details have been added to* **Section 2 of the revised manuscript (pages 7 and 9).**

• In Section 3.1, the description of the DLNN is overly general. Please provide specific architectural details, including: the number of hidden layers and the number of neurons in each layer; input and output dimensions; and whether Dropout, Batch Normalization, or other regularization techniques were employed.

**Response:** *We sincerely thank the reviewer for this insightful comment. In the revised manuscript, Section 3.1 has been thoroughly updated to provide a more detailed and transparent description of the Deep Learning Neural Network (DLNN) model.*

i. *Specifically, we have now explicitly reported the architectural configuration of the DLNN, including the number of hidden layers and the corresponding number of neurons in each layer. The input and output dimensions of the model have also been clearly defined based on the selected soil parameters and the target variable (corrosion depth).*
ii. *In addition, we have clarified the use of activation functions and training settings. Details regarding regularization techniques have also been incorporated. Where applicable, the use (or absence) of Dropout, early stopping, Batch Normalization, and other overfitting control strategies has been explicitly stated and justified to improve reproducibility and transparency.*
iii. *Please see* **pages 11-13** *for more details.*

• In Section 3.2, the mathematical formulation in Equation (2) is incomplete with respect to SVR. The current expression represents only the regression function; it does not include the ε-insensitive loss function, nor does it encompass the optimization problem that defines SVR.

**Response:** *In the revised manuscript,* ***Section 3.2, pages 14-17****, has been carefully updated to provide a complete and rigorous formulation of the Support Vector Regression (SVR) model.*

- *Specifically, the mathematical description has been expanded beyond the regression function to include the* ***ε-insensitive*** *loss function, which defines the tolerance margin within which prediction errors are not penalized. In addition, the full optimization problem underlying SVR has now been incorporated, including the objective function that minimizes model complexity while controlling training error, as well as the associated constraints.*
- *Furthermore, the role of key parameters such as the regularization parameter (C) and the ε-insensitive tube has been clarified to improve the theoretical completeness and interpretability of the model description.*
- *These additions ensure that the SVR formulation is now presented in a complete and standard manner, consistent with established literature, thereby improving the clarity, rigor, and reproducibility of the study.*

• In Section 3.3, the description of XGBoost is somewhat vague and contains several unsubstantiated assertions (e.g., "the preeminent effective model"). Please avoid using overly absolute statements unless they are directly validated within this study or supported by multiple high-quality references.

**Response:** *We sincerely thank the reviewer for this valuable comment. In the revised manuscript, Section 3.3 (****pages 17, and 18****) has been carefully refined to improve clarity, precision, and academic tone.*

1- *Specifically, overly absolute and unsubstantiated statements such as “the preeminent effective model” have been removed or rephrased to maintain a neutral and evidence-based discussion. The description of XGBoost has been revised to focus on its methodological characteristics and documented strengths, such as its efficiency, regularization capability, and suitability for handling nonlinear relationships, while avoiding unsupported generalizations.*
2- *Where appropriate, statements have been either moderated or supported with relevant references to ensure that all claims are properly justified. These revisions enhance the scientific rigor of the manuscript and ensure that conclusions remain aligned with the results obtained in this study rather than relying on broad assertions.*

• In Section 3.4, multiple Linear Regression (MLR) relies on several key assumptions—namely linearity, independence, homoscedasticity, normality of residuals, and the absence of multicollinearity—yet these assumptions were not examined in the text.

**Response:** *We sincerely thank the reviewer for this important observation. In the revised manuscript, Section 3.4 (****page 19****) has been carefully updated to explicitly address and verify the key assumptions underlying the Multiple Linear Regression (MLR) model.*

- *Specifically, the assumption of linearity was examined through residual and scatter plot analyses. The independence of errors was assessed using appropriate statistical diagnostics. Homoscedasticity could be evaluated by analysing the distribution of residuals against fitted values, while the normality of residuals was verified using normal probability (Q–Q) plots and statistical tests. In addition, multicollinearity among the predictor variables was examined using Variance Inflation Factor (VIF) analysis.*
- *These additions have been incorporated into the revised manuscript to ensure that the validity and applicability of the MLR model are properly justified. The updated discussion improves the methodological rigor and provides greater confidence in the reliability of the regression results.*

• In Section 3.5, the manuscript refers to "minority" or "rare" regions of the target variable (dmax), but does not define how to identify these regions.

**Response:** *Thank you for this comment. In our study, “minority” or “rare” regions of d_max_ refer to the low-frequency extreme values in the empirical distribution of the target variable. These correspond to the tail regions of d_max_, which are under-represented in the dataset. This clarification has been added to Section 3.5 in the revised manuscript (****page 21****).*

• In Section 3.6, adopting a single 75/25 train-test split may not provide a robust estimate of performance, particularly given the relatively small size of the dataset (n = 259).

**Response:** *We sincerely appreciate the reviewer’s valuable comment regarding the use of a single 75/25 train–test split.*

*Many previous studies in literature have adopted a similar data partitioning strategy, making it a widely accepted and standard practice for model evaluation. We followed this approach to ensure a fair and consistent comparison with existing research, as also discussed in the discussion section of the manuscript. This split provides a reasonable balance between having sufficient samples for model training while maintaining an independent and reliable test set for evaluation. In addition, using almost the same data split as prior work allows for a more direct and meaningful comparison of our results with those reported in related studies, thereby ensuring a fair and consistent evaluation of our proposed models.*

• In Section 3.7, the descriptions of the metrics appear somewhat disorganized and contain grammatical inconsistencies (e.g., "the parameter measured the error produced by the model").

**Response***: We sincerely thank the reviewer for this valuable comment. Section 3.7 has been revised to improve clarity, grammar, and consistency (****please see page 23****). All metric descriptions have been rewritten in a clearer and more consistent academic style to eliminate ambiguity and enhance readability.*

• In Section 4.1, The process of hyperparameter tuning is not described with sufficient clarity. Please specify the following: tuning strategy (e.g., Grid Search, Random Search, Bayesian Optimization); whether cross-validation was employed during the tuning process; and the objective metrics used to select the optimal parameters.

**Response:** *We have thoroughly clarified our tuning approach in Section 4.1 of the revised manuscript (****please see pages 24-26****) We applied a combined* ***Random Search and trial-and-error method*** *to select the hyperparameters of each model systematically. Random search was used to broadly explore the multi-dimensional parameter space, while trial-and-error allowed us to fine-tune specific ranges to reach optimal model capacity manually. As noted in the first pages of the revised manuscript,* ***no cross-validation was used****. Instead, model capacity and generalisation were balanced by holding out an entirely unseen, independent testing dataset (25%) to verify performance stability against the training set.*

• In Section 4.2, the discussion initially focused on training performance, but this is not a reliable indicator of the model's generalization capability.

**Response:** *The discussion in Section 4.2 has been revised to avoid relying on training results alone and to place greater emphasis on model evaluation using unseen data. The revised section now focuses on performance comparison between training and testing phases to better reflect model generalization capability. Please see pages* ***28 to 30*** *for more details.*

• In Section 4.3, although the DLNN demonstrated superior performance, this came at the expense of its generalization capability. It is recommended that you consider: justifying why the DLNN alone is sufficient; and alternatively, at least providing a comparative analysis against another model (e.g., XGBoost, which offers interpretability).

**Response:** *Thank you for this valuable comment. The sensitivity analysis was conducted exclusively on the DLNN model because it consistently outperformed all other tested algorithms in terms of predictive accuracy, whereas the remaining models did not provide excellent performance; thus, DLNN represents the most appropriate and reliable basis for evaluating feature importance. Since the objective of this study is to assess variable influence within the most accurate predictive framework rather than compare interpretability across different models, cross-model feature importance analysis (e.g., XGBoost) is outside the scope of this work, and this justification has been clarified in the revised manuscript, and the selection of the DLNN approach has been further justified in Section 4.3 (****page 39****).*
