# Supplementary material for: Improving corrosion depth estimation in buried pipelines using SMOTE-integrated machine and deep learning models
Source: PLoS One. 2026 Aug 27;21(8):e0355187. doi: 10.1371/journal.pone.0355187 (PMC13521352; doi:10.1371/journal.pone.0355187)
Supplement: Peer review history

# Peer review: Decision Letter 0

**DOI:** 10.1371/journal.pone.0355187.r001

**Authors:** Xiao Tan

12 May 2026

Dear Dr. Salem,

Thank you for submitting your manuscript to PLOS ONE. After careful consideration, we feel that it has merit but does not fully meet PLOS ONE’s publication criteria as it currently stands. Therefore, we invite you to submit a revised version of the manuscript that addresses the points raised during the review process.

Please submit your revised manuscript by Jun 26 2026 11:59PM. If you will need more time than this to complete your revisions, please reply to this message or contact the journal office at plosone@plos.org. When you're ready to submit your revision, log on to https://www.editorialmanager.com/pone/ and select the 'Submissions Needing Revision' folder to locate your manuscript file.

- A letter that responds to each point raised by the academic editor and reviewer(s). You should upload this letter as a separate file labeled 'Response to Reviewers'.
- A marked-up copy of your manuscript that highlights changes made to the original version. You should upload this as a separate file labeled 'Revised Manuscript with Track Changes'.
- An unmarked version of your revised paper without tracked changes. You should upload this as a separate file labeled 'Manuscript'.

If you would like to make changes to your financial disclosure, please include your updated statement in your cover letter. Guidelines for resubmitting your figure files are available below the reviewer comments at the end of this letter.

If applicable, we recommend that you deposit your laboratory protocols in protocols.io to enhance the reproducibility of your results. Protocols.io assigns your protocol its own identifier (DOI) so that it can be cited independently in the future. For instructions see: https://journals.plos.org/plosone/s/submission-guidelines#loc-laboratory-protocols. Additionally, PLOS ONE offers an option for publishing peer-reviewed Lab Protocol articles, which describe protocols hosted on protocols.io. Read more information on sharing protocols at https://plos.org/protocols?utm_medium=editorial-email&utm_source=authorletters&utm_campaign=protocols.

As the corresponding author, your ORCID iD is verified in the submission system and will appear in the published article. PLOS supports the use of ORCID, and we encourage all coauthors to register for an ORCID iD and use it as well. Please encourage your coauthors to verify their ORCID iD within the submission system before final acceptance, as unverified ORCID iDs will not appear in the published article. *Only*  the individual author can complete the verification step; PLOS staff *cannot*  verify ORCID iDs on behalf of authors.

We look forward to receiving your revised manuscript.

Kind regards,

Xiao Tan, Ph.D.

Academic Editor

PLOS One

Journal Requirements:

When submitting your revision, we need you to address these additional requirements.

1. Please ensure that your manuscript meets PLOS ONE's style requirements, including those for file naming. The PLOS ONE style templates can be found at

https://journals.plos.org/plosone/s/file?id=wjVg/PLOSOne_formatting_sample_main_body.pdf and

https://journals.plos.org/plosone/s/file?id=ba62/PLOSOne_formatting_sample_title_authors_affiliations.pdf

2. Please note that PLOS One has specific guidelines on code sharing for submissions in which author-generated code underpins the findings in the manuscript. In these cases, we expect all author-generated code to be made available without restrictions upon publication of the work. Please review our guidelines at https://journals.plos.org/plosone/s/materials-and-software-sharing#loc-sharing-code and ensure that your code is shared in a way that follows best practice and facilitates reproducibility and reuse.

3. In the online submission form, you indicated that “Data used in this research are available from the corresponding authors upon request.”

All PLOS journals now require all data underlying the findings described in their manuscript to be freely available to other researchers, either 1. In a public repository, 2. Within the manuscript itself, or 3. Uploaded as supplementary information.

This policy applies to all data except where public deposition would breach compliance with the protocol approved by your research ethics board. If your data cannot be made publicly available for ethical or legal reasons (e.g., public availability would compromise patient privacy), please explain your reasons on resubmission and your exemption request will be escalated for approval.

4. If the reviewer comments include a recommendation to cite specific previously published works, please review and evaluate these publications to determine whether they are relevant and should be cited. There is no requirement to cite these works unless the editor has indicated otherwise.

**Additional Editor Comments:**

Thank you for submitting your manuscript to PLOS One. I have completed my evaluation of your manuscript. The reviewers recommend reconsideration of your manuscript following Major Revisions. The reviewers provided constructive comments. The authors are encouraged to carefully address these comments and re-submit a revised manuscript for further consideration. When revising your manuscript, please outline every change made in response to their comments and provide suitable rebuttals for any comments not addressed. Please note that your revised submission may need to be re-reviewed.

[Note: HTML markup is below. Please do not edit.]

Reviewers' comments:

Reviewer's Responses to Questions

**Comments to the Author**

1. Is the manuscript technically sound, and do the data support the conclusions?

Reviewer #1: Yes

Reviewer #2: Yes

**********

2. Has the statistical analysis been performed appropriately and rigorously?

Reviewer #1: Yes

Reviewer #2: Yes

**********

3. Have the authors made all data underlying the findings in their manuscript fully available?

The PLOS Data policy

Reviewer #1: Yes

Reviewer #2: Yes

**********

4. Is the manuscript presented in an intelligible fashion and written in standard English?

Reviewer #1: Yes

Reviewer #2: Yes

**********

Reviewer #1: This paper investigates the use of SMOTE (Synthetic Minority Over‑sampling Technique) to address data imbalance in predicting maximum corrosion depth of buried pipelines. It compares four models (DLNN, XGBoost, SVR, MLR) on original vs. SMOTE‑augmented datasets and reports that DLNN‑SMOTE achieves the best performance (RMSE=0.624, R=0.940). Here are several suggestions for you to revise the manuscript.

1. How did you adapt SMOTE for the continuous target variable (corrosion depth), and what exactly defines a “minority” region in your regression setting?

2. How do you validate that the synthetic samples generated by SMOTE are physically realistic and do not cause overfitting, given your small real dataset (259 samples)?

3. Why did you not include statistical significance tests (e.g., t-test) or other imbalance methods (e.g., SMOGN, weighted loss) as baselines for comparison?

Reviewer #2: Comments:

To the best of the reviewer's knowledge, optical sensing technology for corrosion depth detection is a highly promising technique that can also serve as a reliable data source for machine learning and deep learning. However, the introduction to this field within the paper remains somewhat insufficient. The authors are advised to refer the following references: https://www.mdpi.com/1424-8220/26/9/2641, https://www.sciencedirect.com/science/article/abs/pii/S0030399224000112, https://www.sciencedirect.com/science/article/abs/pii/S0263224125018780.

In Section 2, it is stated that this dataset is derived from "public literature" and a previous technical note [47]; however, the process by which the data was compiled is not explicitly described. Please clarify the following: How were these 259 data points assembled (e.g., were they drawn from multiple studies or a single source)? What criteria were used for the inclusion or exclusion of data? Were any preprocessing steps performed (e.g., normalization, outlier removal, handling of missing data)?

In Section 3.1, the description of the DLNN is overly general. Please provide specific architectural details, including: the number of hidden layers and the number of neurons in each layer; input and output dimensions; and whether Dropout, Batch Normalization, or other regularization techniques were employed.

In Section 3.2, the mathematical formulation in Equation (2) is incomplete with respect to SVR. The current expression represents only the regression function; it does not include the ε-insensitive loss function, nor does it encompass the optimization problem that defines SVR.

In Section 3.3, the description of XGBoost is somewhat vague and contains several unsubstantiated assertions (e.g., "the preeminent effective model"). Please avoid using overly absolute statements unless they are directly validated within this study or supported by multiple high-quality references.

In Section 3.4, multiple Linear Regression (MLR) relies on several key assumptions—namely linearity, independence, homoscedasticity, normality of residuals, and the absence of multicollinearity—yet these assumptions were not examined in the text.

In Section 3.5, the manuscript refers to "minority" or "rare" regions of the target variable (dmax), but does not define how to identify these regions.

In Section 3.6, adopting a single 75/25 train-test split may not provide a robust estimate of performance, particularly given the relatively small size of the dataset (n = 259).

In Section 3.7, the descriptions of the metrics appear somewhat disorganized and contain grammatical inconsistencies (e.g., "the parameter measured the error produced by the model").

In Section 4.1, The process of hyperparameter tuning is not described with sufficient clarity. Please specify the following: tuning strategy (e.g., Grid Search, Random Search, Bayesian Optimization); whether cross-validation was employed during the tuning process; and the objective metrics used to select the optimal parameters.

In Section 4.2, the discussion initially focused on training performance, but this is not a reliable indicator of the model's generalization capability.

In Section 4.3, although the DLNN demonstrated superior performance, this came at the expense of its generalization capability. It is recommended that you consider: justifying why the DLNN alone is sufficient; and alternatively, at least providing a comparative analysis against another model (e.g., XGBoost, which offers interpretability).

**********

what does this mean?). If published, this will include your full peer review and any attached files.

If you choose “no”, your identity will remain anonymous but your review may still be made public.

**Do you want your identity to be public for this peer review?**  For information about this choice, including consent withdrawal, please see our Privacy Policy

Reviewer #1: No

Reviewer #2: **Yes:**  Luyang Xu

**********

[NOTE: If reviewer comments were submitted as an attachment file, they will be attached to this email and accessible via the submission site. Please log into your account, locate the manuscript record, and check for the action link "View Attachments". If this link does not appear, there are no attachment files.]

To ensure your figures meet our technical requirements, please review our figure guidelines: https://journals.plos.org/plosone/s/figures

You may also use PLOS’s free figure tool, NAAS, to help you prepare publication quality figures: https://journals.plos.org/plosone/s/figures#loc-tools-for-figure-preparation.

NAAS will assess whether your figures meet our technical requirements by comparing each figure against our figure specifications.

# Author comment: Author response to Decision Letter 1

**DOI:** 10.1371/journal.pone.0355187.r002

11 Jul 2026

Dear Editor,

We sincerely thank you for the opportunity to submit the revised version of our manuscript to your respected journal. The authors would also like to express their gratitude to the reviewers for their valuable time and constructive comments, which have greatly helped improve the quality of the paper. We confirm that we have made our best efforts to address all reviewer comments and improve the manuscript accordingly. In addition, the authors have clearly provided and clarified the data source and codes used in this study.

Below is a point-by-point response to the reviewers’ comments

Reviewer #1: This paper investigates the use of SMOTE (Synthetic Minority Over‑sampling Technique) to address data imbalance in predicting maximum corrosion depth of buried pipelines. It compares four models (DLNN, XGBoost, SVR, MLR) on original vs. SMOTE‑augmented datasets and reports that DLNN‑SMOTE achieves the best performance (RMSE= 0.624, R= 0.940). Here are several suggestions for you to revise the manuscript.

1. How did you adapt SMOTE for the continuous target variable (corrosion depth), and what exactly defines a “minority” region in your regression setting?

Response: Thank you for this comment. In this study, SMOTE was adapted for regression using a polynomial-based SMOTER approach. In regression, the “minority” is not a class but corresponds to under-represented regions of the continuous target variable. In our case, the extreme corrosion depth values are relatively rare and were treated as minority regions based on their low frequency in the target distribution. SMOTE generates synthetic samples in these regions through interpolation between neighboring data points, thereby improving their representation and enhancing model learning. More details have been added to the revised manuscript (Kindly, see pages 20, and 21).

2. How do you validate that the synthetic samples generated by SMOTE are physically realistic and do not cause overfitting, given your small real dataset (259 samples)?

Response: Thank you for your valuable input

a) We have evaluated the physical realism and statistical validity of the generated synthetic samples using a residual- and distribution-based analysis. The results show that key statistical bounds (minimum and maximum corrosion depth) remain unchanged, indicating that no unrealistic values or extrapolation beyond the physical limits of the original dataset were introduced, while only the distributional characteristics (mean and standard deviation) vary slightly due to improved representation of under-represented regions. For more information, please see Page 27, where it has been clarified that the synthetic samples generated remain physically realistic and consistent with the underlying data distribution.

b) We appreciate this important concern regarding the relatively small dataset size (259 samples). To address the risk of overfitting, the data were randomly divided into separate training and testing sets, ensuring that model evaluation was carried out on unseen data (please see Page 21, section 3.6).

c) Furthermore, the Model behavior was then assessed by examining the consistency between training and testing performance. The observed results show stable and comparable behavior across both phases, indicating good generalization ability. Please see Page 31 and Figure 5b for more detailed information.

3. Why did you not include statistical significance tests (e.g., t-test) or other imbalance methods (e.g., SMOGN, weighted loss) as baselines for comparison?

Response: We thank the reviewer for this valuable suggestion.

i. To check whether the performance differences between competing models are statistically significant, we applied the Wilcoxon signed-rank test, as it is a non-parametric method that does not assume normality of the performance distributions. The results confirm that the improvements of the proposed method over baseline models are statistically significant. Please find more information on page 39.

ii. In this regression study, we use SMOTE with polynomial interpolation to generate synthetic samples to address data imbalance. The experiments are conducted within this resampling-based framework and compared against standard regression baselines under the same setting. A limitation of this study is that it does not include other imbalance-aware regression methods such as SMOGN, which could further strengthen the evaluation, and we have highlighted this in the limitations section of the revised manuscript (Please see Page 43). This will be addressed in future work by incorporating and comparing such approaches.

Reviewer #2: Comments:

To the best of the reviewer's knowledge, optical sensing technology for corrosion depth detection is a highly promising technique that can also serve as a reliable data source for machine learning and deep learning. However, the introduction to this field within the paper remains somewhat insufficient. The authors are advised to refer the following references: https://www.mdpi.com/1424-8220/26/9/2641, https://www.sciencedirect.com/science/article/abs/pii/S0030399224000112, https://www.sciencedirect.com/science/article/abs/pii/S0263224125018780.

Response: Thank you for your valuable suggestion. We have revised the introduction to include optical sensing technologies for corrosion depth detection, and we have incorporated the recommended references to highlight their relevance for providing reliable non-destructive data for machine learning and deep learning models. Please see pages 3 and 4 for more information.

In Section 2, it is stated that this dataset is derived from "public literature" and a previous technical note [47]; however, the process by which the data was compiled is not explicitly described. Please clarify the following: How were these 259 data points assembled (e.g., were they drawn from multiple studies or a single source)? What criteria were used for the inclusion or exclusion of data? Were any preprocessing steps performed (e.g., normalization, outlier removal, handling of missing data)?

Response: The authors thank the reviewer for this comment. The dataset used in this study was adopted directly from previous work, where it was originally compiled from field observations across multiple pipeline excavation sites. No additional data sources were incorporated, and no inclusion or exclusion criteria were applied. The dataset contains no missing values, and all available records were retained. Prior to model development, the data were normalized to ensure consistent feature scaling, and no outlier removal was performed. These details have been added to Section 2 of the revised manuscript (pages 7 and 9).

In Section 3.1, the description of the DLNN is overly general. Please provide specific architectural details, including: the number of hidden layers and the number of neurons in each layer; input and output dimensions; and whether Dropout, Batch Normalization, or other regularization techniques were employed.

Response: We sincerely thank the reviewer for this insightful comment. In the revised manuscript, Section 3.1 has been thoroughly updated to provide a more detailed and transparent description of the Deep Learning Neural Network (DLNN) model.

i. Specifically, we have now explicitly reported the architectural configuration of the DLNN, including the number of hidden layers and the corresponding number of neurons in each layer. The input and output dimensions of the model have also been clearly defined based on the selected soil parameters and the target variable (corrosion depth).

ii. In addition, we have clarified the use of activation functions and training settings. Details regarding regularization techniques have also been incorporated. Where applicable, the use (or absence) of Dropout, early stopping, Batch Normalization, and other overfitting control strategies has been explicitly stated and justified to improve reproducibility and transparency.

iii. Please see pages 11-13 for more details.

In Section 3.2, the mathematical formulation in Equation (2) is incomplete with respect to SVR. The current expression represents only the regression function; it does not include the ε-insensitive loss function, nor does it encompass the optimization problem that defines SVR.

Response: In the revised manuscript, Section 3.2, pages 14-17, has been carefully updated to provide a complete and rigorous formulation of the Support Vector Regression (SVR) model.

� Specifically, the mathematical description has been expanded beyond the regression function to include the ε-insensitive loss function, which defines the tolerance margin within which prediction errors are not penalized. In addition, the full optimization problem underlying SVR has now been incorporated, including the objective function that minimizes model complexity while controlling training error, as well as the associated constraints.

� Furthermore, the role of key parameters such as the regularization parameter (C) and the ε-insensitive tube has been clarified to improve the theoretical completeness and interpretability of the model description.

� These additions ensure that the SVR formulation is now presented in a complete and standard manner, consistent with established literature, thereby improving the clarity, rigor, and reproducibility of the study.

In Section 3.3, the description of XGBoost is somewhat vague and contains several unsubstantiated assertions (e.g., "the preeminent effective model"). Please avoid using overly absolute statements unless they are directly validated within this study or supported by multiple high-quality references.

Response: We sincerely thank the reviewer for this valuable comment. In the revised manuscript, Section 3.3 (pages 17, and 18) has been carefully refined to improve clarity, precision, and academic tone.

1- Specifically, overly absolute and unsubstantiated statements such as “the preeminent effective model” have been removed or rephrased to maintain a neutral and evidence-based discussion. The description of XGBoost has been revised to focus on its methodological characteristics and documented strengths, such as its efficiency, regularization capability, and suitability for handling nonlinear relationships, while avoiding unsupported generalizations.

2- Where appropriate, statements have been either moderated or supported with relevant references to ensure that all claims are properly justified. These revisions enhance the scientific rigor of the manuscript and ensure that conclusions remain aligned with the results obtained in this study rather than relying on broad assertions.

In Section 3.4, multiple Linear Regression (MLR) relies on several key assumptions—namely linearity, independence, homoscedasticity, normality of residuals, and the absence of multicollinearity—yet these assumptions were not examined in the text.

Response: We sincerely thank the reviewer for this important observation. In the revised manuscript, Section 3.4 (page 19) has been carefully updated to explicitly address and verify the key assumptions underlying the Multiple Linear Regression (MLR) model.

- Specifically, the assumption of linearity was examined through residual and scatter plot analyses. The independence of errors was assessed using appropriate statistical diagnostics. Homoscedasticity could be evaluated by analysing the distribution of residuals against fitted values, while the normality of residuals was verified using normal probability (Q–Q) plots and statistical tests. In addition, multicollinearity among the predictor variables was examined using Variance Inflation Factor (VIF) analysis.

- These additions have been incorporated into the revised manuscript to ensure that the validity and applicability of the MLR model are properly justified. The updated discussion improves the methodological rigor and provides greater confidence in the reliability of the regression results.

In Section 3.5, the manuscript refers to "minority" or "rare" regions of the target variable (dmax), but does not define how to identify these regions.

Response: Thank you for this comment. In our study, “minority” or “rare” regions of dmax refer to the low-frequency extreme values in the empirical distribution of the target variable. These correspond to the tail regions of dmax, which are under-represented in the dataset. This clarification has been added to Section 3.5 in the revised manuscript (page 21).

In Section 3.6, adopting a single 75/25 train-test split may not provide a robust estimate of performance, particularly given the relatively small size of the dataset (n = 259).

Response: We sincerely appreciate the reviewer’s valuable comment regarding the use of a single 75/25 train–test split.

Many previous studies in literature have adopted a similar data partitioning strategy, making it a widely accepted and standard practice for model evaluation. We followed this approach to ensure a fair and consistent comparison with existing research, as also discussed in the discussion section of the manuscript. This split provides a reasonable balance between having sufficient samples for model training while maintaining an independent and reliable test set for evaluation. In addition, using almost the same data split as prior work allows for a more direct and meaningful comparison of our results with those reported in related studies, thereby ensuring a fair and consistent evaluation of our proposed models.

In Section 3.7, the descriptions of the metrics appear somewhat disorganized and contain grammatical inconsistencies (e.g., "the parameter measured the error produced by the model").

Response: We sincerely thank the reviewer for this valuable comment. Section 3.7 has been revised to improve clarity, grammar, and consistency (please see page 23). All metric descriptions have been rewritten in a clearer and more consistent academic style to eliminate ambiguity and enhance readability.

In Section 4.1, The process of hyperparameter tuning is not described with sufficient clarity. Please specify the following: tuning strategy (e.g., Grid Search, Random Search, Bayesian Optimization); whether cross-validation was employed during the tuning process; and the objective metrics used to select the optimal parameters.

Response: We have thoroughly clarified our tuning approach in Section 4.1 of the revised manuscript (please see pages 24-26) We applied a combined Random Search and trial-and-error method to select the hyperparameters of each model systematically. Random search was used to broadly explore the multi-dimensional parameter space, while trial-and-error allowed us to fine-tune specific ranges to reach optimal model capacity manually. As noted in the first pages of the revised manuscript, no cross-validation was used. Instead, model capacity and generalisation were balanced by holding out an entirely unseen, independent testing dataset (25%) to verify performance stability against the training set.

In Section 4.2, the discussion initially focused on training performance, but this is not a reliable indicator of the model's generalization capability.

Response: The discussion in Section 4.2 has been revised to avoid relying on training results alone and to place greater emphasis on model evaluation using unseen data. The revised section now focuses on performance comparison between training and testing phases to better reflect model generalization capability. Please see pages 28 to 30 for more details.

In Section 4.3, although the DLNN demonstrated superior performance, this came at the expense of its generalization capability. It is recommended that you consider: justifying why the DLNN alone is sufficient; and alternatively, at least providing a comparative analysis against another model (e.g., XGBoost, which offers interpretability).

Response: Thank you for this valuable comment. The sensitivity analysis was conducted exclusively on the DLNN model because it consistently outperformed all other tested algorithms in terms of predictive accuracy, whereas the remaining models did not provide excellent performance; thus, DLNN represents the

Attachment Submitted filename: Response to Reviewers (1).docx

# Peer review: Decision Letter 1

**DOI:** 10.1371/journal.pone.0355187.r003

**Authors:** Xiao Tan

19 Jul 2026

Improving Corrosion Depth Estimation in Buried Pipelines Using SMOTE-Integrated Machine and Deep Learning Models

PONE-D-26-16891R1

Dear Dr. Salem,

We’re pleased to inform you that your manuscript has been judged scientifically suitable for publication and will be formally accepted for publication once it meets all outstanding technical requirements.

Within one week, you’ll receive an e-mail detailing the required amendments. When these have been addressed, you’ll receive a formal acceptance letter and your manuscript will be scheduled for publication.

An invoice will be generated when your article is formally accepted. Please note, if your institution has a publishing partnership with PLOS and your article meets the relevant criteria, all or part of your publication costs will be covered. Please make sure your user information is up-to-date by logging into Editorial Manager at Editorial Manager® and clicking the ‘Update My Information' link at the top of the page. For questions related to billing, please contact billing support.

If your institution or institutions have a press office, please notify them about your upcoming paper to help maximize its impact. If they’ll be preparing press materials, please inform our press team as soon as possible -- no later than 48 hours after receiving the formal acceptance. Your manuscript will remain under strict press embargo until 2 pm Eastern Time on the date of publication. For more information, please contact onepress@plos.org.

Kind regards,

Xiao Tan, Ph.D.

Academic Editor

PLOS One

Additional Editor Comments (optional):

The comments are well addressed and the reviewers believe this manuscript can be accepted in current form.

Reviewers' comments:

Reviewer's Responses to Questions

**Comments to the Author**

Reviewer #1: All comments have been addressed

Reviewer #2: All comments have been addressed

**********

2. Is the manuscript technically sound, and do the data support the conclusions?

Reviewer #1: Yes

Reviewer #2: Yes

**********

3. Has the statistical analysis been performed appropriately and rigorously?

Reviewer #1: Yes

Reviewer #2: Yes

**********

4. Have the authors made all data underlying the findings in their manuscript fully available?

The PLOS Data policy

Reviewer #1: Yes

Reviewer #2: Yes

**********

5. Is the manuscript presented in an intelligible fashion and written in standard English?

Reviewer #1: Yes

Reviewer #2: Yes

**********

Reviewer #1: The authors have replied all my questions. All required questions have been answered and that all responses meet formatting specifications.It is recommended to be accepted.

Reviewer #2: This manuscript is acceptable for publication. The study addresses a relevant research topic and presents its objectives, methodology, results, and conclusions in a clear and logical manner. The experimental design is appropriate, and the analysis supports the main findings. The manuscript is generally well organized, and the writing is easy to follow. While there may be a few minor issues related to language, formatting, or presentation that could be improved during the revision process, these do not affect the overall quality or scientific value of the work. Therefore, I believe the manuscript meets the journal's publication standards and is suitable for acceptance.

**********

what does this mean?). If published, this will include your full peer review and any attached files.

If you choose “no”, your identity will remain anonymous but your review may still be made public.

**Do you want your identity to be public for this peer review?**  For information about this choice, including consent withdrawal, please see our Privacy Policy

Reviewer #1: No

Reviewer #2: No

**********

# Editor report: Acceptance letter

**DOI:** 10.1371/journal.pone.0355187.r004

**Authors:** Xiao Tan

PONE-D-26-16891R1

PLOS One

Dear Dr. Salem,

I'm pleased to inform you that your manuscript has been deemed suitable for publication in PLOS One. Congratulations! Your manuscript is now being handed over to our production team.

At this stage, our production department will prepare your paper for publication. This includes ensuring the following:

* All references, tables, and figures are properly cited

* All relevant supporting information is included in the manuscript submission,

* There are no issues that prevent the paper from being properly typeset

You will receive further instructions from the production team, including instructions on how to review your proof when it is ready. Please keep in mind that we are working through a large volume of accepted articles, so please give us a few days to review your paper and let you know the next and final steps.

Lastly, if your institution or institutions have a press office, please let them know about your upcoming paper now to help maximize its impact. If they'll be preparing press materials, please inform our press team within the next 48 hours. Your manuscript will remain under strict press embargo until 2 pm Eastern Time on the date of publication. For more information, please contact onepress@plos.org.

You will receive an invoice from PLOS for your publication fee after your manuscript has reached the completed accept phase. If you receive an email requesting payment before acceptance or for any other service, this may be a phishing scheme. Learn how to identify phishing emails and protect your accounts at https://explore.plos.org/phishing.

If we can help with anything else, please email us at customercare@plos.org.

Thank you for submitting your work to PLOS One and supporting open access.

Kind regards,

PLOS ONE Editorial Office Staff

on behalf of

Professor Xiao Tan

Academic Editor

PLOS One
